# Point-of-care ultrasound of the heart and lungs in patients with respiratory failure: a pragmatic randomized controlled multicenter trial

**DOI:** 10.1186/s13049-021-00872-8

**Published:** 2021-04-26

**Authors:** M. Riishede, A. T. Lassen, G. Baatrup, P. I. Pietersen, N. Jacobsen, K. N. Jeschke, C. B. Laursen

**Affiliations:** 1 Department of Surgery, Odense University Hospital, 5700 Svendborg, Denmark; 2 Department of Clinical Research, University of Southern Denmark, SDU-Odense, 5000 Odense, Denmark; 3 Department of Internal Medicine & Emergency Medicine (M/FAM), Odense University Hospital, Valdemarsgade 53, 5700 Svendborg, Denmark; 4 OPEN, Open Patient data Explorative Network, Odense University Hospital, 5000 Odense, Denmark; 5 Department of Emergency Medicine, Odense University Hospital, 5000 Odense, Denmark; 6 Department of Respiratory Medicine, Odense University Hospital, 5000 Odense, Denmark; 7 Regional Center for Technical Simulation (TechSim), Odense University Hospital, 5000 Odense, Denmark; 8 Department of Respiratory Medicine, Copenhagen University Hospital, 2650 Hvidovre, Denmark

**Keywords:** Ultrasound, Point–of-care ultrasound, Emergency department, Respiratory disease, Diagnostic accuracy, High acuity

## Abstract

**Background:**

Point-of-care ultrasound is a focus oriented tool for differentiating among cardiopulmonary diseases. Its value in the hands of emergency physicians, with various ultrasound experience, remains uncertain. We tested the hypothesis that, in emergency department patients with signs of respiratory failure, a point-of-care cardiopulmonary ultrasound along with standard clinical examination, performed by emergency physicians with various ultrasound experience would increase the proportion of patients with presumptive diagnoses in agreement with final diagnoses at four hours after admission compared to standard clinical examination alone.

**Methods:**

In this prospective multicenter superiority trial in Danish emergency departments we randomly assigned patients presenting with acute signs of respiratory failure to intervention or control in a 1:1 ratio by block randomization. Patients received point-of-care cardiopulmonary ultrasound examination within four hours from admission. Ultrasound results were unblinded for the treating emergency physician in the intervention group. Final diagnoses and treatment were determined by blinded review of the medical record after the patients´ discharge.

**Results:**

From October 9, 2015 to April 5, 2017, we randomized 218 patients and included 211 in the final analyses. At four hours we found; no change in the proportion of patients with presumptive diagnoses in agreement with final diagnoses; intervention 79·25% (95% CI 70·3–86·0), control 77·1% (95% CI 68·0–84·3), an increased proportion of appropriate treatment prescribed; intervention 79·3% (95% CI 70·3–86·0), control 65·7% (95% CI 56·0–74·3) and of patients who spent less than 1 day in hospital; intervention *n* = 42 (39·6%, 25·8 38·4), control *n* = 25 (23·8%, 16·5–33·0). No adverse events were reported.

**Conclusions:**

Focused cardiopulmonary ultrasound added to standard clinical examination in patients with signs of respiratory failure had no impact on the diagnostic accuracy, but significantly increased the proportion of appropriate treatment prescribed and the proportion of patients who spent less than 1 day in hospital.

**Trial registration:**

https://clinicaltrials.gov/, number NCT 02550184.

**Supplementary Information:**

The online version contains supplementary material available at 10.1186/s13049-021-00872-8.

## Background

Patients admitted with acute signs of respiratory failure are a challenge for emergency physicians and among the most common indications for admission to the emergency department (ED) among adults [1, 2].

Symptoms are caused by a variety of respiratory and circulatory diseases of which some of the most common are exacerbation in chronic lung disease, pneumonia, pulmonary embolism and congestive heart failure [1, 3]. A fast and correct diagnosis can be crucial as vital signs are often close to normal in those potentially life-threatening diagnoses with a substantial risk of ICU admission [3]. Moreover, these patients are among those with the highest over-all 30-day mortality [4].

Over the past decade point-of-care ultrasound (PoCUS) examination in the ED has gained its place in the armamentarium of diagnostic tools [5, 6].

PoCUS of the lungs has proven to be a helpful tool in the assessment of patients with suspected respiratory diseases and has proven to be superior to chest x-ray in the diagnostic assessment of various pulmonary diseases [7–9].

Combined PoCUS of the heart and lungs has significantly improved the diagnostic accuracy of various cardiopulmonary diseases (e.g., pneumonia, pulmonary embolism, or edema) and identified life threatening diseases missed at primary clinical examination [10–12].

Studies have demonstrated that novice sonographers can find pathology with accuracy comparable to expert sonographers and reference standards with only little training [13, 14].

The amount of diagnostic accuracy studies is vast, and we are short of studies of the PoCUS´ impact on patient related outcomes. Results from diagnostic accuracy studies are rarely directly translational to clinical practice, hence we need randomized pragmatic studies to investigate the potential impact of PoCUS in daily clinical practice.

We investigated the hypothesis that adding a cardiopulmonary PoCUS to standard clinical examination of patients admitted to the ED with acute signs of respiratory failure could increase the proportion of patients with a presumptive diagnosis in agreement with final diagnosis (previously described as `*correct presumptive diagnosis´* in the published protocol) at four hours (4 h) after admission when compared to standard clinical examination alone [15].

## Methods

### Study design

This prospective pragmatic randomized semi-blinded, multicenter, superiority trial with a parallel group design and allocation ratio of 1:1 was undertaken in 10 of the 21 EDs in Danish community Hospitals. Three of these EDs were located at university hospitals and seven in secondary or tertiary hospitals. Patients were enrolled from October 9, 2015 to April 5, 2017.

Acute admissions to Danish EDs are to public hospitals and established by the general practitioner or by emergency call. Exceptions are patients suspected of having heart disease who are admitted directly to the cardiology department.

A presumptive diagnosis at admission and at 4 h as well as a treatment plan must ideally be journalized by the emergency physician (EP) within 4 h from the patient’s admission to the ED.

### Inclusion and exclusion

We screened patients admitted to the ED for inclusion. Patients ≥18 years with a primary sign or symptom of respiratory failure of; cough, dyspnea, chest pain, respiratory frequency > 20 or peripheral oxygen saturation < 95% or any combination of these were included upon written informed consent. Exclusion criteria were inability to give written informed consent, PoCUS of the lungs or heart already performed by others than the investigator in relation to the primary examination, or inability to randomize or perform the PoCUS within 4 h from the patient’s admission to the ED.

### Randomization and masking

Randomization numbers were created by a block randomization database (REDCap, OPEN) using permuted blocks of random numbers to ensure equal numbers of patients in both trial arms at each center [16]. The allocation sequence of randomization numbers was generated by a data manager from REDCap OPEN and the project manager, MR, using an online random number service. Randomization numbers were paired with the REDCap OPEN database developed for this trial by MR.

The investigators screened the patients for inclusion at alternating shifts including day and night shifts on all weekdays. Once a patient signed the informed consent, the investigator registered the patient in the trial database that randomly allocated the patient and assigned the patient a unique computerized randomization number. Patients were aware of their group assignment.

The investigator then noted the basic clinical values upon the patient’s admission and performed the PoCUS unaware of the primary presumptive diagnoses. All PoCUS results were entered in the project database.

PoCUS results from patients in the control group remained blinded to the EP, whereas once the investigator had received the primary presumptive diagnoses, he unblinded the intervention groups PoCUS results to the EP both orally and in writing on a paper record marked with the patients ID number and stored it in an accessible briefcase in the ED. The EP was then free to re-evaluate his presumptive diagnoses and treatment according to the ED’s clinical guidelines. The EP was instructed to leave all information of randomization and PoCUS results out of the medical records to ensure blinding of the medical record audit.

In the medical record the investigator only noted the projects identification number and localization of the paper records containing the PoCUS results.

### Procedures

In all patients admitted to the ED the EP assessed the primary presumptive diagnoses using standard methods of diagnostic examination (e.g.*,* clinical examination, blood samples, ECG, chest x-ray) as soon as possible after the patient’s arrival.

Standard diagnostic tests were available within 4 h. Supplementary imaging examinations such as computerized tomography (CT), ultrasonography and echocardiography performed by specialists were available if necessary.

The investigator performed the PoCUS blinded to the EP in both groups. Subsequently, the EP announced his primary presumptive diagnoses to the investigator who then unblinded the PoCUS findings in the intervention group.

The PoCUS was performed within 4 h from the patients´ admission to the ED and consisted of PoCUS of the heart and lungs. The PoCUS protocol was defined as follows:

The PoCUS of the lungs was a modification of the ultrasound protocol used by Laursen et al. originally modified from the principles of lung ultrasound by Volpicelli and Lichtenstein [12, 17, 18]. It was performed as follows: The anterior and lateral part of thorax was divided into a superior and inferior quadrant. Each quadrant represented a zone in which the probe was placed centrally to create a transverse picture of the costae and pleurae. We looked for pleural effusion, interstitial syndrome/pulmonary edema and pneumothorax.

The PoCUS cardiac ultrasound was performed according to the principles described in the international evidence based guideline [19]. The views used were the 4-chamber picture of the heart achieved either from a sub-xiphoid or an apical window. We looked for pericardial effusion, altered left ventricular ejection fraction and right ventricular overload.

There were no regulations in the choice of ultrasound machines or probes used for inclusion as long as the PoCUS was performed with an image quality deemed sufficient for evaluation by the operator.

All PoCUS examinations were performed by investigators who were either specialists or in specialist training and who received patients in the ED on a regular basis. All investigators used PoCUS on a daily basis in their clinical practice but had varying degrees of PoCUS experience. Prior to become an investigator they all received an educational program regarding data collection and PoCUS examination, which was composed by MR and consisted of an e-learning presentation with instructional videos [20]. Then, MR made a 4 h on site presentation comprising the project, the collection and registration of data and an introduction to the project’s PoCUS protocol. Hereafter, MR evaluated each investigator’s PoCUS skills and handling of the project database by hands on and by multiple choice questionnaire tests to ensure that the investigators were familiar with their investigator tasks prior to initiation of inclusion. During the period of inclusion, investigators could take daily contact to the project manager for questions.

The diagnostic criteria for the PoCUS examination are provided in Appended file 1.

### Diagnostic examinations

The primary presumptive diagnoses were registered in the medical record. The EP was free to reassess the presumptive diagnoses, diagnostic tests, and treatment.

The 4 h presumptive diagnosis was the last registered clinical examination made by the EP within 4 h from the patients´ admission to the ED and was assessed by blinded audit of the medical record after the patients discharge from hospital. New diagnostic tests and treatments could be prescribed after the 4 h examination but would then be part of the final diagnosis.

The final diagnosis was assessed by blinded audit of the medical record and included electronic journal data (e.g. clinical, microbiological and biochemical data, and imaging results) and was performed after the patients´ discharge from hospital by two auditors, who independently of each other, set the final diagnosis. In case of discrepancy a third auditor set the final diagnosis. A predefined audit protocol with diagnostic criteria of the most common diagnoses was used (Appended file 2).

### Outcomes

The primary outcome was to assess the percentage of patients with a presumptive diagnosis in agreement with final diagnosis at 4 h after the patient’s admission to the ED. The 4 h cut off was set because various Danish hospitals request that examination, presumptive diagnoses and plan for further treatment are assessed within 4 h from admission to the ED [21].

The secondary outcomes were as follows:

1. Diagnostic accuracy of the primary presumptive diagnoses made upon arrival.
2. The proportion of patients who upon arrival received a primary presumptive diagnosis in agreement with final diagnosis.
3. The proportion of patients who, within 4 h after admission to the ED, is given the appropriate treatment.
4. Time spent in the ED (hours).
5. Time spent in hospital (days).
6. The proportion of patients who were transferred from the ED to the intensive care unit.
7. The proportion of patients who were transferred from the ED to a hospital ward.
8. The proportion of patients who were discharged from the ED and directly to their home.
9. The proportion of patients who were readmitted to hospital within 30 days from discharge.
10. In-hospital mortality.
11. 30-day mortality.

As a negative PoCUS can be found in various pulmonary diseases (e.g. chronic obstructive pulmonary disease (COPD), asthma) we could not compare PoCUS findings directly with final diagnoses and were omitted from the secondary endpoint `*The proportion of PoCUS examinations with a correct diagnostic examination*´ as written in the published protocol [15].

### Unblinding

For safety and ethical reasons the PoCUS findings in the control group were unblinded to the EP in charge of the patient if the PoCUS raised suspicion of a life-threatening condition (e.g. pulmonary edema, pneumothorax, pericardial effusion, or heart failure) [15].

### Statistical analysis

The sample size estimate was based on the results from a previous similar trial where about 65% of the patients had a presumptive diagnosis in agreement with final diagnosis at 4 h after admission to the ED when PoCUS was not used [11]. A clinically significant improvement of the diagnosis by using PoCUS in a multicenter trial was set to be 15%.

To detect a 15% increase in the number of presumptive diagnoses in agreement with final diagnoses, from 65% in the control group to 80% in the intervention group, with an 80% chance for detection, a level of significance of 5% and with an estimated drop out of 6% we had to include 288 patients, 144 patients in each group (intervention/control). Sample size calculations were made with the online database for clinical trials; Sealed Envelope [22].

We used the intention-to-treat method as main comparative analysis on all participants. Descriptive statistics were handled as follows: Categorical data by number and percentages of patients with 95% confidence intervals (CI), continuous data by number of patients (*n*), mean, standard deviation, median, minimum and maximum.

Missing data in the baseline characteristics were handled as simple imputation when represented as dichotomous data. Other missing data were evaluated to be missing at random and was handled by multiple imputations using the Markov Chain Monte-Carlo method including auxiliary variables in the model. We plotted the mean value of the imputed variables in the spot of missing data. The rule of three was used to find 95% CI in categories without events. Categorical endpoints were summarized by numbers and percentages with 95% confidence intervals [23].

We used the Chi [2] test and the Fischer exact test for comparison of proportions expressed as percentages and for the comparison of continuous endpoints we used the Student t test (means) and the Mann Whitney test (medians). A two-sided significance level of 5% was applied to all tests.

We used the diagnoses from blinded audit as reference test for determining the diagnostic accuracy of the presumptive diagnoses established at admission and at 4 h and their 95% confidence interval based at binominal distribution. To assess the interrater reliability of the final diagnoses we used the Cohens kappa coefficient. Data analyses were conducted using STATA Release V. 15.0 (Stata Corp) including professional statistical advice. To assess the time a patient spent in the ED or hospital and the mortality we withdrew data from the Danish National Patient Register (LPR) administered by the Danish Health Data Authority. No data monitoring committee oversaw the trial.

### Role of the funding source

Funders of this trial had no role in the trial design, data collection, data analysis, data interpretation or writing of the report. The corresponding author had full access to all the trial data and had final responsibility for the decision to submit for publication.

## Results

We included 220 patients from October 9, 2015 to April 5, 2017 in 10 hospitals counting three university hospitals and seven tertiary hospitals with a total of 21 investigators. The trial was ended before we reached the predefined sample size as investigators travelled to other departments and the inclusion stagnated. We initially excluded 9 patients: two patient contacts due to double inclusions (their latest inclusions were excluded), one patient who withdrew informed consent, one patient who had no randomization or PoCUS within 4 h from admission and seven patients due to loss to follow up.

**Fig. 1:**
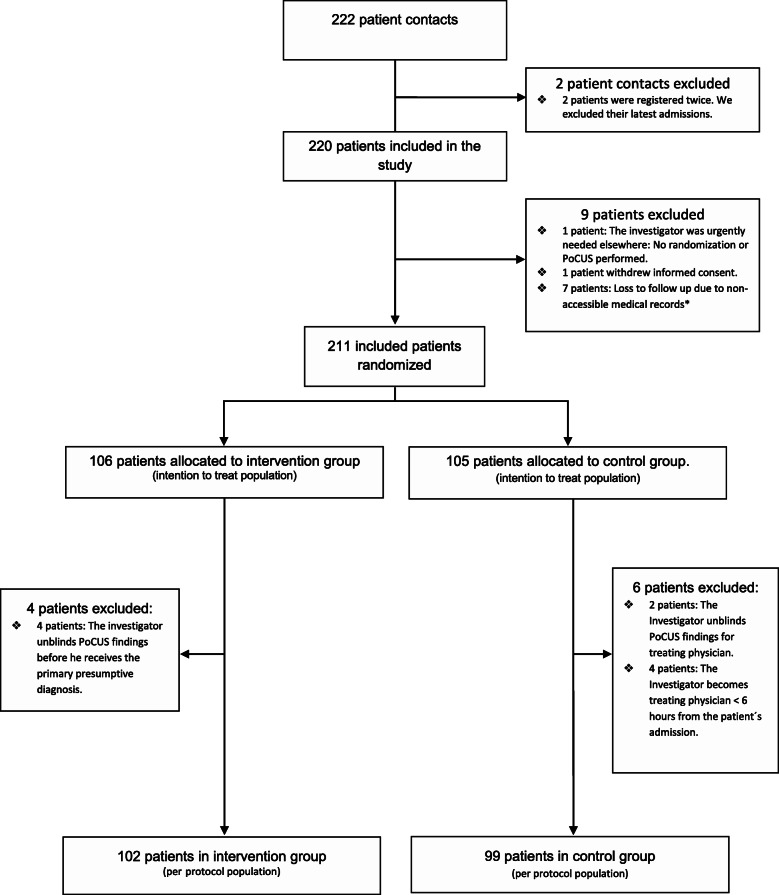
Trial profile (appended). *The 7 randomized patients we lost due to loss of follow up are assessed to be missing at random and were excluded from all analyses as all outcomes were to be calculated by statistic approximation. They were lost as follows: 4 were caused by investigators in two hospitals who withdrew their consent to participation due to too busy working hours in the ED, in 2 the medical records were lost in the transition to a new electronic medical record system, and in 1 an unidentifiable personal ID number was written in the database. Abbreviations: ED (emergency department). PoCUS (Point-of-care ultrasound)

For the intention to treat analyses 211 patients (96%) were randomized: intervention *n* = 106 (50%) and control *n* = 105 (50%). We then excluded 10 patients due to unblinding of the PoCUS results as they were unblinded for the treating physician due to signs of one of the predefined life threatening conditions within 4 h from admission in 6 patients (control *n* = 2, intervention *n* = 4) and as the investigator became treating physician within 6 h from admission in 4 patients (control group). We thereby ended with 201 patients (91%) in the per protocol analyses; intervention *n* = 102 (51%), control *n* = 99 (49%) (Trial profile Fig. 1).

There are no deviations of the PoCUS examination in either group due to adverse events or complications related to the trial and no patients had PoCUS performed in the ambulance prior to admission to the ED. For patient baseline characteristics see Table 1. For PoCUS findings see Appended file 3.

**Table 1:** Baseline characteristics of the intention to treat population

	Intervention group (***n*** = 106)	Control group (***n*** = 105)
**Age** (years; median (IQR)	68 (51–79)	69 (62–79)
**Sex**		
Male	62 (58%)	46 (44%)
Female	44 (42%)	59 (56%)
**Smoking status**		
Never smoked	30 (28%)	25 (24%)
Current smoker	27 (25%)	12 (11%)
Previous smoker	24 (23%)	37 (35%)
Unknown status	25 (24%)	31 (30%)
**Medical history**		
Apoplexy	9 (8%)	7 (7%)
Coronary artery disease	17 (16%)	16 (15%)
Heart failure	13 (12%)	14 (13%)
Arterial hypertension	24 (23%)	22 (21%)
Thromboembolic disease	11 (10%)	5 (5%)
Chronic obstructive pulmonary disease	33 (31%)	32 (30%)
Asthma	6 (6%)	6 (6%)
Other pulmonary or pleural lung disease	11 (10%)	9 (9%)
Diabetes mellitus	8 (8%)	17 (16%)
Chronic kidney disease	8 (8%)	8 (8%)
Other medical disease	28 (26%)	36 (34%)
Psychiatric disorder	7 (7%)	4 (4%)
**Medication at admission**		
Β-blockers	28 (26%)	23 (22%)
Diuretics	38 (36%)	49 (47%)
Nitrates	9 (8%)	14 (13%)
Angiotensin-converting-enzyme inhibitor or angiotensin-receptor blocker	26 (25%)	38 (36%)
Digoxin	4 (4%)	5 (5%)
Calcium-channel blockers	16 (15%)	21 (20%)
Aspirin	16 (15%)	18 (17%)
Inhaled bronchodilators	39 (37%)	45 (43%)
Inhaled corticosteroids	21 (20%)	31 (30%)
Oral corticosteroids	10 (9%)	17 (16%)
Antibiotics	18 (17%)	17 (16%)
Anticoagulants	29 (27%)	36 (34%)
Immunosuppressive medication	5 (5%)	2 (2%)
Other medication	19 (18%)	24 (23%)
**Vital signs at admission** mean, (min-max)		
Respiratory rate (breaths per min)	21 (12–44)	22 (12–40)
Saturation (%)	94 (55–100)	95 (74–100)
- of these patients n received oxygen supply (l/min)	23 (22%)	29 (28%)
o 1–3 l/min n (%)	15 (65%)	24 (83%)
o 4–6 l/min n (%)	8 (35%)	4 (14%)
o > 6 l/min n (%)	0	1 (3%)
Systolic blood pressure (mmHg)	137 (80–224)	137 (94–212)
Diastolic blood pressure (mmHg)	78 (32–123)	78 (47–111)
Heart rate (beats per minute)	90 (52–150)	86 (40–144)
Temperature (°C)	37.1 (35.2–39.5)	37.2 (35.0–39.8)
Blood glucose	6.8 (4.1–19.9)	6.6 (1.0–22.4)
Glasgow coma scale score^a^	15 (9–15)	15 (14–15)
**Patients´ signs and symptoms upon admission**		
Cough	33 (31%)	43 (41%)
Dyspnoea	90 (85%)	85 (81%)
Chest pain	28 (26%)	28 (27%)
Respiration rate > 20 breaths per min	33 (30%)	30 (29%)
Peripheral saturation < 95%	30 (28%)	24 (23%)
None of the above	0	0
**Ultrasound examination already performed in ambulance**	0	0
**Patients severity score upon admission**^b^		
- I (Red)	5 (5%)	1 (1%)
- II (Orange)	29 (27%)	36 (34%)
-	49 (46%)	42 (40%)
- III (Yellow) - IV (Green)	23 (22%)	27 (26%)

Data are number (n), (%), mean (SD) or a median (IQR), unless otherwise indicated

Data are not available for all randomized patients. Missing data are handled by multiple imputation for continuous data and simple imputation when binominary

^a^We found GCS 9 in one patient admitted with exacerbation in terminal COPD. Pt was immediately treated with NIV with effect, replied relevant on questions and signed informed consent. The remaining of the included patients had GCS from 14 to 15

^b^The severity score is made according to the Danish Emergency Process Triage (DEPT) criteria used for patients with acute illness. The severity score is assessed by measuring the patients´ vital parameters (e.g. BP, HR, GCS). `I (Red)´ is the most severe condition

Final diagnoses were assessed by the two auditors with an overall agreement of 93·18% (kappa 0·58) ranging from 81·04–100% (kappa − 0·02–1·0) within the specific diagnoses. A detailed description of the interrater reliability of the audit is provided in Appended file 4. The most common final diagnoses in the intervention and control group were; pneumonia (25%/ 32%), exacerbation of COPD (25%/27%) and systolic heart failure (16%/21%). The proportion of presumptive diagnoses in agreement with final diagnoses at 4 h in the intervention group is increased compared to the control group for the diagnoses of exacerbation of COPD (89%/75%), pulmonary edema (53%/33%) and para-pneumonic effusion (77%/38%) but with overlapping CIs. The proportion of correctly diagnosed pneumonia is equal among the groups (88%/88%) (Tables 2, 3).

**Table 2:** Final diagnoses and the proportion of presumptive diagnoses in agreement with final diagnoses at 4 h

	Diagnoses in the intervention group (***n =*** 106)		Diagnoses in the control group (***n =*** 105)	
	Final diagnoses, n (%).	Number of 4 h presumptive diagnoses in agreement with final diagnoses, n (%).	Final diagnoses, n (%).	Number of 4 h presumptive diagnoses in agreement with final diagnoses, n (%).
**Lungs**				
Exacerbation of COPD	27 (25%)	24 (89%)	28 (27%)	21 (75%)
Asthma with exacerbation	2 (2%)	0	3 (3%)	2 (67%)
Exacerbation in ILD	3 (3%)	1 (33%)	5 (5%)	0
Pneumonia	26 (25%)	23 (88%)	34 (32%)	30 (88%)
Pulmonary edema	17 (16%)	9 (53%)	6 (6%)	2 (33%)
Parapneumonic effusion	14 (13%)	10 (77%)	14 (13%)	5 (38%)
Empyema	1 (1%)	0	0	0
Pulmonary embolism	3 (3%)	3 (100%)	2 (2%)	2 (100%)
Pneumothorax	0	0	1 (1%)	1 (100%)
**Heart**				
Systolic heart failure	17 (16%)	13 (76%)	22 (21%)	13 (62%)
Non-systolic heart failure	5 (5%)	3 (60%)	1 (1%)	1 (100%)
Acute myocardial infarction	1 (1%)	0	2 (2%)	1 (50%)
**Miscellaneous**				
Anemia	5 (5%)	1 (20%)	6 (6%)	0
Malignancy	8 (8%)	2 (25%)	19 (18%)	5 (26%)
No diagnostic criteria met	33 (31%)	19 (58%)	35 (33%)	21 (60%)

Intention to treat population

Abbreviations: *n* number of patients, *COPD* Chronic obstructive pulmonary disease, *ILD* interstitial lung disease

**Table 3:** Diagnostic accuracy of emergency physicians´ 4 h presumptive diagnoses compared to final diagnoses

Diagnoses	Final diagnosis positive / 4 h positive		Sensitivity% (95% CI)		Specificity% (95% CI)		PPV % (95% CI)		NPV % (95% CI)	
	Interv.	Control	Interv.	Control	Interv.	Control	Interv.	Control	Interv.	Control
**COPD with exacerbation**	27/29	28/24	89 (71–98)	75 (55–89)	94 (86–98)	96 (89–99)	83 (64–94)	88 (68–97)	96 (89–99)	91 (83–97)
**Asthma with exacerbation**	2/2	3/3	0 (0–84)	67 (9–99)	98 (93–100)	99 (94–100)	0 (0–84)	67 (9–99)	98 (93–100)	99 (94–100)
**Interstitial lung disease**	3/1	5/2	33 (1–91)	0 (0–52)	100 (97–100)	98 (93–100)	100 (3–100)	0 (0–84)	98 (93–100)	95 (88–98)
**Pneumonia**	26/34	34/40	89 (70–98)	88 (73–97)	86 (77–93)	86 (76–93)	68 (50–83)	75 (59–87)	96 (88–99)	94 (85–98)
**Pulmonary edema**	17/10	6/4	53 (28–77)	33 (4–78)	99 (94–100)	98 (93–100)	90 (56–100)	50 (7–93)	92 (84–96)	96 (90–99)
**Para-pneumonic effusion**	14/14	14/7	71 (42–92)	36 (13–65)	96 (89–99)	98 (92–100)	71 (42–92)	71 (29–96)	96 (89–99)	91 (83–96)
**Pleural empyema**^a^	1/0	0/0	–	–	–	–	–	–	–	–
**Pulmonary embolism**	3/11	2/7	100 (29–100)	100 (16–100)	92 (85–97)	96 (89–98)	27 (6–61)	29 (4–71)	100 (96–100)	100 (96–100)
**Pneumothorax**^a^	0/0	1/3	–	100 (3–100)	–	98 (93–100)	–	33 (1–91)	–	100 (96–100)
**Systolic heart failure**	17/16	22/18	77 (50–93)	59 (36–79)	97 (91–99)	94 (87–98)	81 (54–96)	72 (47–90)	95 (89–99)	90 (81–95)
**Non-systolic heart failure**	5/6	1/2	60 (15–95)	100 (3–100)	97 (92–99)	99 (95–100)	50 (12–88)	50 (1–99)	98 (93–100)	100 (97–100)
**Acute myocardial infarction**	1/2	2/9	0 (0–98)	50 (1–99)	98 (93–100)	92 (85–97)	0 (0–84)	11 (0–48)	99 (95–100)	99 (94–100)
**Anemia**	5/1	6/1	20 (1–72)	0 (0–46)	100 (96–100)	99 (95–100)	100 (3–100)	0 (0–98)	96 (91–99)	94 (88–98)
**Malignancy**	8/2	19/7	25 (3–65)	26 (9–51)	94 (96–100)	98 (92–100)	100 (16–100)	71 (29–96)	94 (88–98)	86 (77–92)
**Other diagnoses**	33/29	35/31	58 (39–75)	60 (42–76)	86 (76–93)	86 (75–93)	66(46–82)	68 (49–83)	83(71–90)	81 (70–89)

Intention to treat population

Intervention (*n* = 106), control (*n* = 105)

Abbreviation: *Interv* Intervention

^a^Too few ratings to perform diagnostic accuracy calculations

No difference was found in the proportion of patients who received a presumptive diagnosis in agreement with final diagnosis at arrival; intervention group, *n* = 65 (61·32%; 95% CI 51·59–70·23) versus control group, *n* = 65 (61·90%; 95% CI 52·12–70·80), (*p* = 0·93) nor at 4 h after admission to the ED; intervention group, *n* = 84 (79·25%; 70·32–86·02) versus control group, *n* = 81 (77·14%; 68·00–84·28) (*p =* 0·71). However, the proportion of patients who had an appropriate treatment prescribed at 4 h was significantly larger in the intervention group, *n* = 84 (79·25%; 95% CI 70·32–86·02) than in the control group *n* = 69 (65·71%; 95% CI 55·99–74·27) (*p* = 0·03) with an absolute increase of 13·5% (Table 4).

**Table 4:** Primary and secondary outcomes for the intention to treat population

	Intervention group (***n*** = 106) n (%; 95% CI)	Control group (***n*** = 105) n (%; 95% CI)	***P*** value	Absolute effect % (95% CI)	Relative effect (95% CI)
**PRIMARY ENDPOINT**					
**4 h after admission to the ED**					
Proportion of patients with presumptive diagnoses in agreement with final diagnoses	84 (79.3%; 70.3–86.0)	81 (77.1%; 68.0–84.3)	0.71	2.1% (−9.0–13.2)	1.03(0.89–1.18)
**SECONDARY ENDPOINTS**					
**4 h after admission to the ED**					
Proportion of patients with appropriate treatment ordered ^a^	84 (79.3%; 70.3–86.0)	69 (65.7%; 56.0–74.3)	0.03	13.5% (1.6–25.5)	1.21 (1.02–1.43)
**After primary assessment in the ED**					
Proportion of patients with presumptive diagnoses in agreement with final diagnoses	65 (61.3%; 51.6–70.2)	65 (61.9%; 52.1–70.8)	0.93	−0.5% (−13.7–12.5)	0.99(0.80–1.23)
**Specific treatment prescribed within 4 h from admission to the ED**					
Oxygen	33 (31.1%; 22.5–40.0)	37 (35.2%; 26. 2–45.2)	0.53	−4.1% (−16.8–8.6)	0.88 (0.60–1.30)
NIV/CPAP	3 (2.80%; 0.9–8.4)	0 (0%; 0–3.5^¥^)	0.08	2.8% (−0.3–6.0)	–
Respirator	0 (0%; 0–3.4^¥^)	0 (0%; 0–3.5^¥^)	–	0	–
Bronchodilators	26 (24.5%; 16.5–34.0)	20 (19.1%; 12.0–27.9)	0.34	5.5% (−5.6–16.6)	1.29 (0.77–2.16)
Systemic steroids	24 (22.6%; 14.9–31.9)	21 (20.0%; 12.8–28.9)	0.64	2.6% (−8.4–13.7)	1.13 (0.67–1.90)
Antibiotics	30 (28.3%; 19.9–38.2)	38 (36.2%; 27.0–46.1)	0.22	−7.9% (−20.5–4.7)	0.78 (0.53–1.16)
Fluids i.v.	15 (14.2%; 7.7–22.0)	25 (23.8%; 16.0–33.1)	0.07	−9.7% (− 20.2–0.8)	0.59 (0.33–1.06)
Diuretics	13 (12.3%; 7.0–20.8)	12 (11.4%; 6.0–19.1)	0.85	0.8% (−7.9–9.6)	1.07 (0.51–2.24)
Antiarrythmics	4 (3.8%; 1.1–9.7)	3 (2.9%; 0.6–8.1)	0.71	0.9% (−3.9–5.7)	1.32 (0.30–5.76)
Vasoconstrictors	0 (0%; 0–3.4^¥^)	0 (0%; 0–3.5^¥^)	–	0	–
Anticoagulants	10 (9.4%; 4.8–17-3)	13 (12.4%; 6.8–20.2)	0.49	−2.9% (−11.4–5.5)	0.76 (0.35–1.66)
Therapeutic centesis ^b^	3 (2.8%; 0.6–8.4)	3 (2.9%; 0.6–8.1)	0.99	−0.0% (−4.5–4.5)	0.99 (0.20–4.80)
Others	8 (7.6%; 3.4–14.9)	6 (5.7%; 2.1–12.0)	0.59	1.8 (−4.9–8.5)	1.32 (0.47–3.68)
**Supplementary diagnostic tests ordered within 4 h from admission to the ED**					
Ultrasound of the lungs ^c^	34 (32.1%; 23.3–41.8)	10 (9.5%; 4.7–16.8)	0.0001	22.6% (12.0–33.1)	3.37 (1.76–6.46)
X-ray of the thorax	85 (80.2%; 71.3—87.2)	90 (85.7%; 77.5–91.8)	0.29	−5.5% (−15.6–4.6)	0.94 (0.83–1.06)
CT of the thorax	10 (9.4%; 4.6–16.7)	9 (8.6%; 4.0–15.6)	0.83	0.9% (−6.9–8.6)	1.10 (0.47–2.60)
MR of the thorax	0 (0%; 0–3.4¥)	0 (0%; 0–3.5¥)	–	0	–
Diagnostic centesis^b^	1 (0.9%; 0.0–5.1)	2 (1.9%; 2.3–6.7)	0.56	−1.0% (−4.2–2.2)	0.50 (0.05–5.58)
Echocardiography by cardiologist	15 (14.2.%; 8.1–22.3)	19 (18.1%; 11.3–26.8)	0.44	−3.9% (−13.9–6.0)	0.78 (0.42–1.45)
Ultrasound of the deep veins ^d^	2 (1.90%; 0.2–6.6)	0 (0%; 0–3.5¥)	0.16	1.9% (−0.7–4.5)	–
Other diagnostic tests	24 (22.6%; 15.1–31.8)	12 (11.4%; 6.0–19.1)	0.02	11.2% (1.2–21.2)	1.98 (1.05–3.75)
**Time spent in the ED (hours)**					
< 1	24 (22.6%; 15.6–31.7)	28 (26.7%; 19.0–36.1)	0.50	−4.0% (−15.6–7.6)	0.85 (0.53–1.36)
1–2	20 (18.9%; 12.4–27.6)	24 (22.9%; 15.7–32.0)	0.48	−4.0% (−14.9–7.0)	0.83 (0.49–1.40)
3–4	23 (21.7%; 14.8–30.7)	18 (17.1%; 11.0–25.7)	0.40	4.6% (−6.1–15.2)	1.3 (0.73–2.20)
5–8	15 (14.2%; 8.6–22.3)	17 (16.2%; 10.2–24.7)	0.68	−2.0% (−11.7–7.6)	0.87 (0.46–1.66)
9–24	10 (9.4%; 5.1–16.8)	5 (4.8%; 2.0–11.1)	0.19	4.7% (−2.2–11.6)	1.98 (0.70–5.60)
25–48	8 (7.6%; 3.8–14.5)	5 (4.8%; 2.0–11.1)	0.40	2.8% (−3.7–9.3)	1.58 (0.54–4.69)
> 48	6 (5.7%; 2.5–12-2)	9 (8.6%; 4.5–15.8)	0.60	−2.0% (−8.7–4.8)	0.74 (0.27–2.07)
**Time spent in hospital (days)**					
< 1	42 (39.6%; 25.8 38.4)	25 (23.8%; 16.5–33.0)	0.01	15.8% (3.4–28.2)	1.66 (1.10–2.52)
1	14 (13.2%; 12.1–22.3)	21 (20.0%; 13.3–28.9)	0.18	−6.8% (−16.8–3.2)	0.66 (0.36–1.23)
2	8 (7.6%; 5.0–12.6)	9 (8.6%; 4.5–15.8)	0.78	−1.0% (−8.4–6.3)	0.88 (0.35–2.19)
3	11 (10.4%; 5.7–13.7)	8 (7.6%; 3.8–14.6)	0.48	2.8% (−5.0–10.5)	1.36 (0.57–3.25)
4–7	16 (15.1%; 14.2–24.9)	24 (22.9%; 15.7–32.0)	0.15	−7.8% (−18.3–2.8)	0.66 (0.37–1.17)
> 7	15 (14.2%; 11.3–21.2)	18 (17.4%; 11.0–25.7)	0.55	−3.0% (−12.8–6.8)	0.83 (0.44–1.55)
**Itinerary: After discharge from the ED patients were:**					
Transferred to the ICU	0 (0%; 0–3.4¥)	1 (1%; 0.1–6.6)	0.31	−1.0% (−0.3–0.0)	0
Transferred to a hospital ward	52 (49.0%; 39.5–58.6)	52 (49.5%; 39.9–59.1)	0.95	−0.5% (−14.0–13.0)	0.99 (0.75–1.30)
Sent home	54 (50.9%; 41.4–60.5)	52 (49.5%; 39.9–59.1)	0.84	−1.42% (− 12.1–14.9)	1.03 (0.79–1.35)
**Readmission**					
Patients readmitted ≤30 days from discharge	23 (21.7%; 14.8–30.7)	23 (21.9%; 14.9–31.0)	0.97	−0.26% (−11.3–10.9)	0.99 (0.59–1.65)
**Mortality**					
In hospital	2 (1.9%; 0.2–6.6)	4 (3.8%; 1.0–9.5)	0.40	−1.9% (−6.4–2.6)	0.49 (0.92–2.64)
30 day mortality	2 (1.9%; 0.2–6.6)	7 (6.7%; 2.7–13.3)	0.09	−4.8% (− 10.2–0.6)	0.28 (0.06–1.33)

**Abbreviations:** *n* number of patients, *NIV* Non-invasive ventilation, *CPAP* Continuous positive airway pressure, *ED* emergency department, *ICU* intensive care unit. One-sided 97.5% confidence interval

^a^According to local treatment guidelines for the diseases in question

^b^Pleura-, thoraco-, cardio-centesis

^c^Performed by a radiologist or ultrasonographer certified in lung ultrasound

^d^Performed by radiologist or certified ultrasonographer

In the intervention group, a significantly increased proportion of patients had supplementary diagnostic tests performed within the first 4 h, and the most frequent diagnostic test was PoCUS of the lungs performed by a physician specialized in lung ultrasound; intervention *n* = 34 (32·1%; 23·3–41·8), control *n* = 10 (9·5%; 4·7–16·8) (*p* = 0·0001).

Moreover, a significantly increased proportion of patients in the intervention group spent less than 1 day in hospital, *n* = 42 (39·6%; 25·8–38·4) compared to the control group *n* = 25 (23·8%; 16·5–33·0) (*p =* 0·01) clarified by an absolute increase of 15·8%. Aside from that we found no significant changes in the proportion of time spent in the ED or in hospital, in the patients´ itinerary after discharge from the ED or in the proportion of readmissions and mortality (Table 4).

The diagnostic accuracy of the 4 h presumptive diagnoses compared to final diagnoses are listed in Table 2 and added contingency tables in Appended file 5. For `Other diagnoses´ than final diagnoses, see Appended file 6. Causes of death in patients who died within 30 days from admission are summarized in Appended file 7. For per protocol analysis, see Appended file 8.

No adverse events related to the PoCUS were observed.

## Discussion

To our knowledge, no similar randomized pragmatic multicenter trials of adding PoCUS to standard clinical examination have been performed in adult ED patients admitted with signs of acute respiratory failure in which they have been looking at both the diagnostic accuracy, the proportion of appropriate treatment prescribed and the length of stay.

In this trial we found that adding PoCUS to standard clinical examination in adult patients with acute signs of respiratory failure led to an insignificant, absolute increase of 2·1% (*p =* 0·71) in the proportion of patients with presumptive diagnoses in agreement with final diagnoses at 4 h after admission to the ED. We observed a significant, absolute increase of 13·5% (*p =* 0·03) in the proportion of patients who had appropriate treatment prescribed within 4 h from admission and of patients who stayed less than 1 day in hospital (Table 4).

We find our patient population comparable to similar studies. The basic characteristics of our study groups were highly consistent regarding demographic and clinical characteristics, with only a slight imbalance in the distribution of gender and they had a comparable combination of vital signs and medical history upon admission [11, 24].

The insignificant increase of the presumptive diagnoses in agreement with final diagnoses at 4 h is surprising. The high 4 h diagnostic accuracy found in the control group was unexpected when compared to a similar single center trial, which reported an absolute increase of presumptive diagnoses in agreement with final diagnoses of 24·3% (from 63·7% to 88·0, 95% CI 15·0–33·1) and of appropriate treatment prescribed of 21·2% (from 56·7 to 78%, 95% CI 10·8–30·9) at 4 h after the patients admission (*p* < 0·0001). They added cardiopulmonary PoCUS to standard clinical assessment performed by a single physician specialized in PoCUS [11]. However, this does not explain the high 4 h diagnostic accuracy we found in both groups nor the significant increase of appropriate treatment prescribed that we found in the intervention group [11].

Instead, the high diagnostic accuracy at 4 h can be due to the augmented focus on getting more specialist doctors in front in the first critical hours from the patients´ admission to the ED, which may have increased the diagnostic accuracy in general.

The significant increase of 13.5% in the proportion of patients in the intervention group who, at 4 h from admission, had an appropriate treatment prescribed despite an insignificant difference in diagnostic accuracy was similar to the single center study by Laursen et al. who found an absolute increase of 21·2% (Table 4) [11].

Laursen et al. found that the diagnostic tests prescribed within the first 4 h in the intervention group had a higher proportion of tests in which the suspected diagnoses were confirmed, but that the proportion of diagnostic tests prescribed evened out when compared throughout the entire hospital stay [11]. These results indicate that the implementation of PoCUS leads to faster execution of diagnostic tests earlier in the hospital stay to confirm or reject a presumptive diagnosis and could be a contributory cause to the significant increase in the proportion of appropriate prescribed treatment at 4 h in the intervention group. Finally, combined with the significant increase in the proportion of intervention patients who spent less than 1 day in hospital, these results indicate that the PoCUS has a positive impact on several clinical outcome parameters.

Apart from the above mentioned, we found no difference in the patients´ itinerary, time spent in hospital or in the rate of readmissions or mortality as found by Laursen et al. ^11^ The length of time spent in the ED or in hospital is often fairly short hence, larger studies are needed to assess whether PoCUS has a benefit on these parameters.

In our study population we find a low number of severely ill patients (Table 1) and surprisingly few with pulmonary edema, pulmonary embolism and acute myocardial infarction despite a greater proportion of patients with systolic heart failure than found in similar trials [11, 24, 25]. We cannot rule out that a selection bias has taken place where severely ill patients have been deselected due to the more cumbersome process of including these patients in a study trial.

We may have reduced the number of final diagnoses of pulmonary edema (Table 2) as the investigators´ PoCUS findings of pulmonary edema did not overrule a negative chest x-ray in the diagnostic criteria despite that PoCUS of the lungs has shown higher diagnostic accuracy. And as we did not implement pro-brain natriuretic peptide (BNP) in the diagnostic criteria, we may have reduced the number of final diagnoses of cardiac pathology [9]. The proportion of patients with pulmonary embolism or AMI might be low due to the Danish emergency department setting that bypasses the ED and directs these patients straight to the department of cardiology or to outpatient clinics, and because the prevalence of cardiopulmonary diseases is known to vary across studies despite similar inclusion criteria (Table 2) [11, 24, 26]. Moreover, we limited the subjective bias from the blinded audit of the final diagnoses by predefined diagnostic criteria based on internationally accepted guidelines. However, the kappa agreement between the two auditors was only weak to moderate according to Cohen’s guidelines, probably because the auditors had to agree on all diagnoses to obtain agreement or because the kappa statistic values tend to underestimate the agreement in situations with high inter-observer agreement (Appended file 4). However, the inter-observer variability reflects daily clinical dilemmas where a final clear diagnosis is not always possible to establish. Hence, we find no reason to believe that the manner of evaluating the final diagnoses plays a significant role in the results as it has been tested in similar studies [11, 27, 28]. This study indicates that cardiopulmonary PoCUS in the hands of non-specialist sonographers can impact patient treatment, underlined by studies of steep learning curves which demonstrates that novice sonographers can find pathology comparable to gold standards [13, 14]. The pragmatic multicenter trial design increases the generalizability of the results.

Nevertheless, the study has limitations. Firstly, we never reached sample size (inclusion: 220 versus sample size: 288) which might have caused the lack of statistically significant results. Second, enrolment only took place when the investigators were present in the ED, which might have caused bias. Third, the broad inclusion criteria prompted the inclusion of a large number of patients with diseases others than cardiopulmonary. Compared to other studies we ended up with several patients who had pathology that the PoCUS has no diagnostic impact on which may have caused a reduction in the absolute effect of our outcome parameters [11]. Fourth, our diagnostic criteria may have been too strict as we refrained from applying the BTS criteria that indicates that pneumonia can be diagnosed if there is “*no other explanation for the illness, which is treated as community acquired pneumonia (CAP) with antibiotics*” (Appended file 2), and from the investigators positive PoCUS findings of pneumonia, despite that PoCUS of the lungs has proven to be superior to chest x-ray in diagnosing pneumonia. This is emphasized by a higher proportion of diagnoses of pneumonia at 4 h than at final diagnoses in both groups as well as a lack of increase in the diagnostic accuracy (Table 3) [8, 29]. Fifth, the general absence of a CT-thorax or complete echocardiography in the assessment of the final diagnoses is a weakness in the standard criteria of the final cardiopulmonary diagnoses and may have reduced the amount of significant findings. But, as it applies to all included patients we believe it to be of less importance.

Our study was not designed robust enough to enlighten the actual impact that PoCUS might have on acute as well as on hard clinical outcomes as patient morbidity, mortality and time spent in hospital. Larger studies are needed to specify the recommendations and the selection of patients whom would benefit the most from this diagnostic test as it has substantial healthcare and socioeconomic potential.

## Conclusion

PoCUS in combination with standard clinical examination, performed in patients admitted to the ED with acute signs of respiratory failure, did not increase the proportion of patients who received a presumptive diagnosis in agreement with final diagnosis within 4 h from admission. However, PoCUS in combination with standard clinical examination was superior to standard clinical examination alone as it increased the proportion of patients, who had appropriate treatment prescribed within 4 h from admission and who spent less than 1 day in hospital.

## Supplementary Information

**Additional file 1.**

**Additional file 2.**

**Additional file 3.**

**Additional file 4.**

**Additional file 5.**

**Additional file 6.**

**Additional file 7.**

**Additional file 8.**

## Data Availability

The datasets analyzed during the current study are available from the corresponding author on reasonable request.

## Abbreviations

CI: Confidence interval
COPD: Chronic obstructive pulmonary disease
CT: Computerized tomography
ED: Emergency department
EP: Emergency physician
n: Number of patients
PoCUS: Point-of-care ultrasound
4 h: 4 h
