# Supplementary material for: Point-of-care ultrasound of the heart and lungs in patients with respiratory failure: a pragmatic randomized controlled multicenter trial
Source: Scand J Trauma Resusc Emerg Med. 2021 Apr 26;29:60. doi: 10.1186/s13049-021-00872-8 (PMC8073910; doi:10.1186/s13049-021-00872-8)
Supplement: Supplementary file 1 — Additional file 1. [file 13049_2021_872_MOESM1_ESM.docx]

**Additional file 1**

**Diagnostic criteria of the point-of-care ultrasound examination**

These following sonographic definitions and diagnostic criteria are inspired from the sonographic definitions and diagnostic criteria by Laursen et al. and from international evidence-based recommendations for point-of-care ultrasound (PoCUS) of the lungs ^1 2^. Changes include a reduction in diagnostic measurements in order to make it apply to our study. Thereby the investigators only measure the pericardial effusion but not the ventricle wall or diameter nor the tricuspid annular plane systolic excursion (TAPSE). Small changes have been made in the estimation of the ejection fraction. The diagnostic criteria of the cardiac and pulmonary ultrasound examination have been reduced.

**Point-of-care ultrasound of the heart**

PoCUS of the heart is performed according to principles described in the international evidence based guideline ^3^. Only regular measurements are applied to the presence of pericardial effusion. The remaining criteria are estimated by eyeballing. The following criteria are used as we looked for:

**Pericardial effusion**

Presence of an echo-free zone of > 0,5cm (measured in the diastole) and which separates the pericardium from the heart.

**Left ventricle ejection fraction**

The left ventricle ejection fraction is estimated by “eye-balling” and sub classified into:

- Mild left ventricular systolic heart failure: Ejection fraction: 45-65 %
- Moderate left ventricular systolic heart failure: Ejection fraction 31-44%
- Severe left ventricular systolic heart failure: Ejection fraction ≤ 30%

**Right ventricular overload**

- Dilation of the right ventricle; the diameter of right ventricle ≥ left ventricle diameter

**Point-of-care ultrasound of the lungs**

As previously stated PoCUS of the lungs is a modification of the ultrasound protocol used by Laursen et al. ^4^. It is originally modified from the principles of lung ultrasound by Volpicelli and Lichtenstein ^5 6^. The definitions and diagnostic criteria for the ultrasound findings are modified from Laursen et al. ^1^ who based his modifications on the EFSUMB course book ^7^

Modifications consist of:

1) Reduction in subdivisions of ultrasound findings.

2) A redefinition in the severity of pleural effusion.

To perform a complete ultrasound examination of the lungs we used four windows on each hemi-thorax: two anterior and two laterals.

We looked for:

**Pleural effusion**

The diagnosis of pleural effusion is based on the presence of any echo-free zone separating the visceral and parietal pleura.

**Interstitial syndrome/pulmonary edema**

We use the classification of diffuse interstitial syndrome (IS) which is a pattern that can be seen in a variety of diseases as for example pulmonary edema, adult respiratory distress syndrome and interstitial lung disease.

Diffuse IS: Presence of multiple (≥3) B-lines in an intercostal space in at least 2 lateral or anterior areas on each side.

**Pneumothorax**

Confirmed pneumothorax: Area with the absence of lung sliding, lung point and B-lines but with the presence of a lung point in an adjacent area.

Suspected pneumothorax: Absence of lung sliding, B-lines, lung pulse and a lung point.

**Image Quality**

Upon review by the specialists in ultrasound the image quality will be graded on a scale from one to five. Each number on the scale is defined as:

1. Poor image quality: it is not possible to recognize any anatomical structures.
2. Impaired image quality: some anatomical structures can be visualized, but it is still not possible to diagnose or exclude any pathology.
3. Suboptimal image quality: some anatomical structures can be visualized, and it is possible to diagnose or exclude rough pathology.
4. Acceptable image quality: all relevant anatomical structures and any potential pathology can be visualized, but still the resolution of the image is not perfect.
5. Excellent image quality: all relevant anatomical structures and any potential pathology can be visualized and the resolution of the picture is near perfect.

**References**

1. Laursen CB, Sloth E, Lassen AT, et al. Focused sonographic examination of the heart, lungs and deep veins in an unselected population of acute admitted patients with respiratory symptoms: a protocol for a prospective, blinded, randomised controlled trial. *BMJ open* 2012;2(3) doi: 10.1136/bmjopen-2012-001369 [published Online First: 2012/06/01]

2. Volpicelli G, Elbarbary M, Blaivas M, et al. International evidence-based recommendations for point-of-care lung ultrasound. *Intensive care medicine* 2012;38(4):577-91. doi: 10.1007/s00134-012-2513-4 [published Online First: 2012/03/07]

3. Via G, Hussain A, Wells M, et al. International evidence-based recommendations for focused cardiac ultrasound. *Journal of the American Society of Echocardiography : official publication of the American Society of Echocardiography* 2014;27(7):683.e1-83.e33. doi: 10.1016/j.echo.2014.05.001 [published Online First: 2014/06/22]

4. Laursen CB, Sloth E, Lassen AT, et al. Point-of-care ultrasonography in patients admitted with respiratory symptoms: a single-blind, randomised controlled trial. *The Lancet Respiratory medicine* 2014;2(8):638-46. doi: 10.1016/s2213-2600(14)70135-3 [published Online First: 2014/07/08]

5. Volpicelli G, Mussa A, Garofalo G, et al. Bedside lung ultrasound in the assessment of alveolar-interstitial syndrome. *The American journal of emergency medicine* 2006;24(6):689-96. doi: 10.1016/j.ajem.2006.02.013 [published Online First: 2006/09/21]

6. Lichtenstein D. General Ultrasound in the Critically Ill . *Berlin Heidelberg, Germany: Springer-Verlag* 2007

7. Mathis G. EFSUMB Course Book. 2010(<http://issuu.com/efsumb/docs/coursebook-chestsono_ch17?e=3336122/6603975):Chp> 17. UL Chest.
