# Supplementary material for: Point-of-care ultrasound of the heart and lungs in patients with respiratory failure: a pragmatic randomized controlled multicenter trial
Source: Scand J Trauma Resusc Emerg Med. 2021 Apr 26;29:60. doi: 10.1186/s13049-021-00872-8 (PMC8073910; doi:10.1186/s13049-021-00872-8)
Supplement: Supplementary file 2 — Additional file 2. [file 13049_2021_872_MOESM2_ESM.docx]

**Additional file 2**

**Diagnostic criteria of blinded audit and final diagnoses**

**Blinded audit of final diagnoses**

The final diagnoses in both groups were established partly by blinded audit and partly by the result of diagnostic imaging. Two physicians, independent of each other, performed the audit of the patients’ entire hospital stay. The physicians used the diagnostic criteria listed below. For each patient both physicians filled out a registration form containing the diagnostic criteria below and registered which criteria / diagnoses were met and which were not met. Once the two physicians, independent of each other, agreed on a diagnosis, it was accepted as the final diagnosis. However, upon disagreement of the final diagnosis a third physician made the final conclusion by a consensus agreement on the final diagnosis.

**Blinded audit diagnostic criteria**

The following criteria for blinded audit are an extension of the ones used in the study by Laursen et al [1].

**CEREBRAL DISEASE**

**Cerebral stroke**

Patients diagnosed with cerebral stroke according to the diagnostic criteria of the AHA/SAS 2013 [2].

All of the following criteria must be present:

**-** Symptoms of CNS infarction = / > 24 hours or until death.

**-** Verification by CT-cerebrum/MR-cerebrum/pathology/other objective evidence.

**Cerebral hemorrhagia**

All of the following criteria must be present [2]

- Rapidly evolving neurological symptoms.
- Verified by CT-cerebrum/MR-cerebrum/pathology or other objective evidence.

**LUNG DISEASE**

**COPD and COPD with exacerbation**

All of the following criteria must be present:

- Patient diagnosed with COPD according to GOLD guidelines[3].
- Symptoms compatible with COPD exacerbation with a worsening in one or more of the following: dyspnoea, sputum production or cough.

**Asthma**

- Patient diagnosed with asthma according to GINA guidelines[4].

**Asthma with exacerbation**

All of the following criteria must be present:

- Patient diagnosed with asthma according to GINA guidelines[4].
- Symptoms compatible with asthma exacerbation with a progressive worsening in one or more of the following: shortness of breath, wheezing, coughing or chest tightness.
- Clinical examination with signs compatible with asthma exacerbation (prolonged expiration, wheezing, PEF lower than personal best which demonstrates a change from the patient’s usual status that is sufficient to require a change in treatment.

**Interstitial lung disease**

The patient has, either previously or during the hospital stay, been seen by a specialist in pulmonary medicine and diagnosed as having an interstitial lung disease according to the guidelines from the Danish Society of Respiratory Medicine end the European Respiratory Society guidelines [5, 6].

**Pneumonia**

The diagnostic criteria are based on the BTS definition of community acquired pneumonia [7]. The exception being that the BTS criteria which states that “No other explanation for the illness, which is treated as CAP with antibiotics” is omitted. Pneumonia is defined as the presence of all of the following:

- Symptoms of an acute lower respiratory tract illness (cough and at least one other lower respiratory tract symptom).
- New focal chest signs on examination.
- At least one systemic feature (either a symptom complex of sweating, fevers, shivers, aches and pains and/or temperature of 38°C or more).
- New radiographic shadowing for which there is no other explanation (eg. not pulmonary edema or infarction).

**Pulmonary edema**

2 or more of the following criteria must be present:

- Signs of pulmonary edema defined as the presence of increased respiratory rate, hypoxemia and auscultation with bilateral lung crepitation.
- Radiological examination with signs of pulmonary edema (chest x-ray, CT of the chest)
- Elevated B-type natriuretic peptide (BNP) or elevated N-terminal fragment BNP (NT-proBNP).

**Pleural effusion**

- Pleural effusion diagnosed by either radiological examination: chest x-ray/CT of the chest/ultrasound by radiologist/thoracocentesis.

**Parapneumonic effusion**

All of the following must be present:

- Pleural effusion diagnosed by either radiological examination: chest x-ray/CT of the chest/ultrasound by radiologist/thoracocentesis.
- Co-existing infection in the lung on the same side as the effusion (e.g. diagnostic criteria for

pneumonia met).

- Diagnostic criteria for empyema not met.

**Empyema**

The presence of purulent / turbid / cloudy pleural fluid or a positive Gram stain / culture of pleural fluid.

**Pulmonary embolism**

Pulmonary embolism diagnosed by:

- CT of the chest
- MR of the chest
- Angiography
- Ventilation perfusion-scan (examination with a high risk of pulmonary embolism).

**Pneumothorax**

Pneumothorax diagnosed by a radiological examination (chest x-ray/CT of the chest).

**HEART FAILURE**

Diagnosed when either the criteria for systolic or non-systolic heart failure is met.

**Systolic heart failure**

The criteria used are based on Task Force for Diagnosis and Treatment of Acute and Chronic Heart Failure 2008 of European Society of Cardiology guidelines [8]. All of the following criteria must be present:

- Symptoms typical of heart failure (breathlessness at rest or on exercise, fatigue, tiredness, ankle swelling).
- Signs typical of heart failure (tachycardia, tachypnoea, pulmonary rales, pleural effusion, raised jugular venous pressure, peripheral edema, hepatomegaly).
- Objective evidence of a functional abnormality of the heart at rest, defined as echocardiography with reduced left ventricle ejection fraction (< 45%).
- Diagnostic echocardiography performed by a cardiologist.

**Non-systolic heart failure**

The criteria are based on recommendations by European Society of Cardiology Study Group on Diastolic Heart[9]. All of the following criteria must be present:

- Signs or symptoms of congestive heart failure: Exertional dyspnea, eventually objective evidence by reduced peak exercise oxygen consumption, orthopnea, gallop sounds, lung crepitation, pulmonary edema.
- Normal or mildly reduced left ventricular systolic function
- Evidence of abnormal left ventricular relaxation, filling, diastolic dispensability and diastolic stiffness (slow isovolumetric left ventricular relaxation and / or slow early left ventricular filling and/or reduced left ventricular diastolic distensability and/or increased left ventricular chamber or muscle stiffness).
- Diagnostic echocardiography performed by a cardiologist.

**Myocardial infarction**

Diagnosed according to the consensus document of The Joint European Society of Cardiology / American College of Cardiology Committee for the Redefinition of Myocardial Infarction [10] .

Criteria for acute, evolving or recent MI: Either one of the following criteria satisfies the diagnosis for an acute, evolving or recent MI:

1) Typical rise and gradual fall (troponin) or more rapid rise and fall (CK-MB) of biochemical markers of myocardial necrosis with at least one of the following:

- Ischemic symptoms
- Development of pathologic Q waves on the ECG
- ECG changes indicative of ischemia (ST segment elevation or depression)
- Coronary artery intervention (e.g. coronary angioplasty).

2) Pathologic findings of an acute MI.

Criteria for established MI: Any one of the following criteria satisfies the diagnosis for established MI:

(1) Development of new pathologic Q waves on serial ECGs. The patient may or may not remember previous symptoms. Biochemical markers of myocardial necrosis may have normalized, depending on the length of time that has passed since the infarct developed.

(2) Pathologic findings of a healed or healing MI: Diagnosis confirmed by echocardiography performed by a cardiologist.

**Pericardial effusion**

Diagnosis confirmed by echocardiography performed by a cardiologist.

**Valvular heart disease**

Diagnosed according to the ECS 2012 guidelines [11]. Either one of the following criteria satisfies the diagnosis:

- EKKO
- cardiac MR
- cardiac catheterisation
- multi-detector CT including measurements of the aortic annulus.

**Infective endocarditis**

Diagnosed according to the modified Duke criteria [12, 13].

**Cardiac arrhythmia**

Diagnosed with ECG /telemetry.

**Chest pain**

Diagnosed by subjective sensation of chest pain where no other pathology is found and myocardial infarction is ruled out.

**ABDOMINAL DISEASE**

**Ileus**

One of the following must be present:

- Diagnosed by X-ray/CT of the abdomen
- Diagnosed at surgical procedure

**Appendicitis**

One of the following must be present:

- Diagnosed at surgical procedure
- Diagnosed by autopsy (pathological description)
- Diagnosed by abdominal CT

**Dissection or aneurism of the abdominal aorta**

One of the following must be present:

- Diagnosed by CT/MR/Ultrasound of the abdomen
- Diagnosed by autopsy (pathological description)

**Free fluid abdomen**

One of the following must be present:

- Diagnosed by X-ray/CT/MR/Ultrasound of the abdomen

**Pancreatitis**

Diagnosed by:

- Medical history, clinical examination
- Blood test or CT/MR/Ultrasound of the abdomen
- Histopathological description

**Kidney stone**

One of the following must be present:

- Diagnosed by CT/Ultrasound of the abdomen
- Diagnosed by surgical procedure
- Diagnosed by autopsy (pathological description)

**ORTHOPEDIC DISEASE**

**Fracture**

One of the following must be present:

- Diagnosed by X-ray/CT/MR
- Diagnosed at surgical procedure

**Luxation**

One of the following must be present:

- Diagnosed by X-ray/CT/MR
- Diagnosed at surgical procedure

**Orthopedic injuries**

Orthopedic injuries which are not fractures or luxation.

**OTHER DISEASES OR SYMPTOMS**

**Deep vein thrombosis**

Diagnoses confirmed by one of the following:

- Ultrasound performed by a radiologist
- Intravenous venography (conventional or CT)

**Anemia**

Diagnosed according to the WHO diagnostic criteria [14].

Anemia is subdivided into light, moderate and severe, according to the following:

Men:

- Light anemia: 6 mmol/l < Hb < 8.1 mmol/l

- Moderate anemia: 4 mmol/l < Hb < 6.1 mmol/l

- Severe anemia: Hb < 4.1 mmol/l

Women:

- Light anemia: 6 mmol/l < Hb < 7.5 mmol/l

- Moderate anemia: 4 mmol/l < Hb < 6.1 mmol/l

- Severe anemia: Hb < 4.1 mmol/l

**Malignancy**

Diagnosis confirmed by either histology or cytology. The extent of the disease estimated by radiological examination (e.g. computed tomography, magnetic resonance imaging, positron emission tomography, chest x-ray).

**Poisoning**

Diagnosed according to the guidelines for laboratory analyses [15] and confirmed by:

- Documented exposure or clinical presentation in concordance with toxicological exposure
- Biochemical or toxicological analyses of blood or urine

**Fainting/dizziness/discomfort**

Diagnosed by:

- Testified fainting/subjective sensation of vertigo/subjective feeling of discomfort.
- No other pathological explanation upon medical examination and clinical tests.

**Infection with extra-pulmonary focus**

Is defined according to the criteria for specific types of infections in the acute setting defined by The Centers of Disease Control and Prevention and National Healthcare Safety Network [16]

**No diagnostic criteria met**

If the patient does not fulfill any of the above mentioned criteria final diagnosis is made by the auditor. The auditor reaches the final diagnosis by clinical judgement based upon the patient’s previous medical history and all information from the hospital.

**References**

1 Laursen CB, Sloth E, Lassen AT *et al*: Focused sonographic examination of the heart, lungs and deep veins in an unselected population of acute admitted patients with respiratory symptoms: a protocol for a prospective, blinded, randomised controlled trial. *BMJ Open* 2012; 2;(3).

2 Sacco RL, Kasner SE, Broderick JP *et al*: An updated definition of stroke for the 21st century: a statement for healthcare professionals from the American Heart Association/American Stroke Association. *Stroke* 2013; 44;(7):2064-89.

3 Global Strategy for the Diagnosis, Management and Prevention of COPD, Global Initiative for Chronic Obstructive Lung Disease (GOLD) 2015. Available from: <http://www.goldcopd.org/>.

4 Global Strategy for Asthma Management and Prevention, Global Initiative for Asthma (GINA) 2015. Available from: <http://www.ginasthma.org/>.

5 <http://www.lungemedicin.dk/foreningen.html> DSoRM.

6 Raghu G, Collard HR, Egan JJ *et al*: An official ATS/ERS/JRS/ALAT statement: idiopathic pulmonary fibrosis: evidence-based guidelines for diagnosis and management. *Am J Respir Crit Care Med* 2011; 183;(6):788-824.

7 Lim WS, Baudouin SV, George RC *et al*: BTS guidelines for the management of community acquired pneumonia in adults: update 2009. *Thorax* 2009; 64 Suppl 3:iii1-55.

8 Dickstein K, Cohen-Solal A, Filippatos G *et al*: ESC guidelines for the diagnosis and treatment of acute and chronic heart failure 2008: the Task Force for the diagnosis and treatment of acute and chronic heart failure 2008 of the European Society of Cardiology. Developed in collaboration with the Heart Failure Association of the ESC (HFA) and endorsed by the European Society of Intensive Care Medicine (ESICM). *Eur J Heart Fail* 2008; 10;(10):933-89.

9 How to diagnose diastolic heart failure. European Study Group on Diastolic Heart Failure. *Eur Heart J* 1998; 19;(7):990-1003.

10 Myocardial infarction redefined--a consensus document of The Joint European Society of Cardiology/American College of Cardiology Committee for the redefinition of myocardial infarction. *Eur Heart J* 2000; 21;(18):1502-13.

11 Vahanian A, Alfieri O, Andreotti F *et al*: [Guidelines on the management of valvular heart disease (version 2012). The Joint Task Force on the Management of Valvular Heart Disease of the European Society of Cardiology (ESC) and the European Association for Cardio-Thoracic Surgery (EACTS)]. *G Ital Cardiol (Rome)* 2013; 14;(3):167-214.

12 Durack DT, Lukes AS, Bright DK: New criteria for diagnosis of infective endocarditis: utilization of specific echocardiographic findings. Duke Endocarditis Service. *Am J Med* 1994; 96;(3):200-9.

13 DUKE criteria for infective endocarditis. New criteria. [*http://wwwmedcalccom/endocarditishtml*](http://wwwmedcalccom/endocarditishtml).

14 World Health Organization (2008). Worldwide prevalence of anaemia 1993–2005. Geneva: World Health Organization. ISBN 9789241596657.

15 Thompson JP, Watson ID, Thanacoody HK *et al*: Guidelines for laboratory analyses for poisoned patients in the United Kingdom. *Ann Clin Biochem* 2014; 51;(Pt 3):312-25.

16 Horan TC, Andrus M, Dudeck MA: CDC/NHSN surveillance definition of health care-associated infection and criteria for specific types of infections in the acute care setting. *Am J Infect Control* 2008; 36;(5):309-32.
