# Supplementary material for: Point-of-care ultrasound of the heart and lungs in patients with respiratory failure: a pragmatic randomized controlled multicenter trial
Source: Scand J Trauma Resusc Emerg Med. 2021 Apr 26;29:60. doi: 10.1186/s13049-021-00872-8 (PMC8073910; doi:10.1186/s13049-021-00872-8)
Supplement: Supplementary file 3 — Additional file 3. [file 13049_2021_872_MOESM3_ESM.docx]

**Additional file 3**

**Point-of-care ultrasound findings and the consequences hereof in the intention to treat population.**

| **Findings and consequences** | **Intervention (n=106)**  **n (%)** | **Control (n=105)**  **n (%)** |
| --- | --- | --- |
| **Pathological PoCUS of the lungs** | **56 (53)** | **49 (47)** |
| Pneumothorax | 0 | 1 |
| Pleural effusion | 29 | 21 |
| Interstitial syndrome | 36 | 36 |
| Other * | 1 | 1 |
| **Pathological PoCUS of the heart** | **39 (37)** | **37 (35)** |
| Pericardial effusion | 1 | 0 |
| Right ventricular overload | 9 | 12 |
| Reduced ejection fraction | 39 | 36 |
| **Consequence of PoCUS of the heart:** |  |  |
| Acute ECHO prescribed | 6 | 2 |
| **Acute ECHO performed by:**  Investigator (certified in ECHO)  Summoned specialist (certified in ECHO) | 2  4 | 1  1 |
| Change of diagnosis | 1 | 0 |
| Change of treatment | 2 | 0 |
| Patient transferred to other department | 1 | 1 |

Abbreviation: ECHO (echocardiography) PoCUS (Point-of-care ultrasound)

*No description of positive pathological findings.
