# Supplementary material for: Point-of-care ultrasound of the heart and lungs in patients with respiratory failure: a pragmatic randomized controlled multicenter trial
Source: Scand J Trauma Resusc Emerg Med. 2021 Apr 26;29:60. doi: 10.1186/s13049-021-00872-8 (PMC8073910; doi:10.1186/s13049-021-00872-8)
Supplement: Supplementary file 4 — Additional file 4. [file 13049_2021_872_MOESM4_ESM.docx]

**Additional file 4**

**Interrater reliability of final diagnoses assessed by blinded audit of the medical records by investigator 1 and 2 in both intervention and control group (N=211).**

| **Diagnosis** | **Agreement (%)** | **Expected agreement (%)** | **Kappa** |
| --- | --- | --- | --- |
| COPD | 90.52 | 66,44 | 0.72 |
| Asthma | 99.05 | 98.12 | 0,50 |
| Interstitial lung disease | 97,63 | 95,80 | 0,44 |
| Pneumonia | 81,04 | 70,87 | 0.35 |
| Pulmonary edema | 91,94 | 84,00 | 0.50 |
| Parapneumonic effusion | 96,68 | 96,71 | -0,01 |
| Empyema | 100 | 99.06 | 1.0 |
| Pulmonary embolism | 100 | 95.37 | 1.0 |
| Pneumothorax** | 100 | 99.06 | 1.0 |
| Systolic heart failure | 92,42 | 79,83 | 0,62 |
| Non-systolic heart failure | 95.73 | 95,82 | -0,02 |
| Acute myocardial infarction | 99,05 | 97,19 | 0.66 |
| Anemia | 93.84 | 92,95 | 0,13 |
| Malignancy | 86,73 | 79,08 | 0,37 |
| Other diagnosis | 72,99 | 49,68 | 0,46 |

| **Diagnosis** | **Agreement (%)** | **Expected agreement** | **Kappa** |
| --- | --- | --- | --- |
| All diagnoses in total | 93.18 | 83.61 | 0.58 |
