# Supplementary material for: Point-of-care ultrasound of the heart and lungs in patients with respiratory failure: a pragmatic randomized controlled multicenter trial
Source: Scand J Trauma Resusc Emerg Med. 2021 Apr 26;29:60. doi: 10.1186/s13049-021-00872-8 (PMC8073910; doi:10.1186/s13049-021-00872-8)
Supplement: Supplementary file 5 — Additional file 5. [file 13049_2021_872_MOESM5_ESM.zip › Additional file 5a_ Diagnostic accuracy 4h_Int_cr1.docx]

**Additional file 5a**

**Diagnostic accuracy of 4 hour diagnoses. Intervention group.**

Correlation and diagnostic accuracy between investigators´ 4 hour presumptive diagnoses and final diagnoses (reference standards). Intention to treat population.

**Intervention group**

COPD in exacerbation

| **4 hour diagnosis** | **Final diagnosis** | | **Total** |
| --- | --- | --- | --- |
|  | **1** | **0** |  |
| **1** | 24 | 5 | 29 |
| **0** | 3 | 74 | 77 |
| **Total** | 27 | 79 | 106 |
|  | | | |
| Sensitivity % (95% CI) | Specificity % (95% CI) | PPV % (95% CI) | NPV % (95% CI) |
| 89 (71-98) | 94 (86-98) | 83 (64-94) | 96 (89-99) |

Asthma in exacerbation

| **4 hour diagnosis** | **Final diagnosis** | | **Total** |
| --- | --- | --- | --- |
|  | **1** | **0** |  |
| **1** | 0 | 2 | 2 |
| **0** | 2 | 102 | 104 |
| **Total** | 2 | 104 | 106 |
|  | | | |
| Sensitivity % (95% CI) | Specificity % (95% CI) | PPV % (95% CI) | NPV % (95% CI) |
| 0 (0-84) | 98 (93-100) | 0 (0-84) | 98 (93-100) |

Interstitial lung disease

| **4 hour diagnosis** | **Final diagnosis** | | **Total** |
| --- | --- | --- | --- |
|  | **1** | **0** |  |
| **1** | 1 | 0 | 1 |
| **0** | 2 | 103 | 105 |
| **Total** | 3 | 103 | 106 |
|  | | | |
| Sensitivity % (95% CI) | Specificity % (95% CI) | PPV % (95% CI) | NPV % (95% CI) |
| 33 (1-91) | 100 (97-100) | 100 (3-100) | 98 (93-100) |

Pneumonia

| **4 hour diagnosis** | **Final diagnosis** | | **Total** |
| --- | --- | --- | --- |
|  | **1** | **0** |  |
| **1** | 23 | 11 | 34 |
| **0** | 3 | 69 | 72 |
| **Total** | 26 | 80 | 106 |
|  | | | |
| Sensitivity % (95% CI) | Specificity % (95% CI) | PPV % (95% CI) | NPV % (95% CI) |
| 89 (70-98) | 86 (77-93) | 68 (50-83) | 96 (88-99) |

Pulmonary edema

| **4 hour diagnosis** | **Final diagnosis** | | **Total** |
| --- | --- | --- | --- |
|  | **1** | **0** |  |
| **1** | 9 | 1 | 10 |
| **0** | 8 | 88 | 96 |
| **Total** | 17 | 89 | 106 |
|  | | | |
| Sensitivity % (95% CI) | Specificity % (95% CI) | PPV % (95% CI) | NPV % (95% CI) |
| 53 (28-77) | 99 (94-100) | 90 (56-100) | 92 (84-96) |

Para-pneumonic effusion

| **4 hour diagnosis** | **Final diagnosis** | | **Total** |
| --- | --- | --- | --- |
|  | **1** | **0** |  |
| **1** | 10 | 4 | 14 |
| **0** | 4 | 88 | 92 |
| **Total** | 14 | 92 | 106 |
|  | | | |
| Sensitivity % (95% CI) | Specificity % (95% CI) | PPV % (95% CI) | NPV % (95% CI) |
| 71 (42-92) | 96 (89-99) | 71 (42-92) | 96 (89-99) |

Pulmonary empyema *

| 4 hour diagnosis | Final diagnosis | | Total |
| --- | --- | --- | --- |
|  | 1 | 0 |  |
| 1 | 0 | 0 | 0 |
| 0 | 1 | 105 | 106 |
| Total | 1 | 105 | 106 |
|  | | | |
| Sensitivity % (95% CI) | Specificity % (95% CI) | PPV % (95% CI) | NPV % (95% CI) |
| - | - | - | - |

Pulmonary emboli

| **4 hour diagnosis** | **Final diagnosis** | | **Total** |
| --- | --- | --- | --- |
|  | **1** | **0** |  |
| **1** | 3 | 8 | 11 |
| **0** | 0 | 95 | 95 |
| **Total** | 3 | 103 | 106 |
|  | | | |
| Sensitivity % (95% CI) | Specificity % (95% CI) | PPV % (95% CI) | NPV % (95% CI) |
| 100 (29-100) | 92 (85-97) | 27 (6-61) | 100 (96-100) |

Pneumothorax *

| **4 hour diagnosis** | **Final diagnosis** | | **Total** |
| --- | --- | --- | --- |
|  | **1** | **0** |  |
| **1** | 0 | 0 | 0 |
| **0** | 0 | 106 | 106 |
| **Total** | 0 | 106 | 106 |
|  | | | |
| Sensitivity % (95% CI) | Specificity % (95% CI) | PPV % (95% CI) | NPV % (95% CI) |
| **-** | - | - | - |

Systolic heart failure

| **4 hour diagnosis** | **Final diagnosis** | | **Total** |
| --- | --- | --- | --- |
|  | **1** | **0** |  |
| **1** | 13 | 3 | 16 |
| **0** | 4 | 86 | 90 |
| **Total** | 17 | 89 | 106 |
|  | | | |
| Sensitivity % (95% CI) | Specificity % (95% CI) | PPV % (95% CI) | NPV % (95% CI) |
| 77 (50-93) | 97 (91-99) | 81 (54-96) | 96 (89-99) |

Non-systolic heart failure

| **4 hour diagnosis** | **Final diagnosis** | | **Total** |
| --- | --- | --- | --- |
|  | **1** | **0** |  |
| **1** | 3 | 3 | 6 |
| **0** | 2 | 98 | 100 |
| **Total** | 5 | 101 | 106 |
|  | | | |
| Sensitivity % (95% CI) | Specificity % (95% CI) | PPV % (95% CI) | NPV % (95% CI) |
| 60 (15-95) | 97 (92-99) | 50 (12-88) | 98 (93-100) |

Acute myocardial infarction

| **4 hour diagnosis** | **Final diagnosis** | | **Total** |
| --- | --- | --- | --- |
|  | **1** | **0** |  |
| **1** | 0 | 2 | 2 |
| **0** | 1 | 103 | 104 |
| **Total** | 1 | 105 | 106 |
|  | | | |
| Sensitivity % (95% CI) | Specificity % (95% CI) | PPV % (95% CI) | NPV % (95% CI) |
| 0 (0-98) | 98 (93-100) | 0 (0-84) | 99 (95-100) |

Anemia

| **4 hour diagnosis** | **Final diagnosis** | | **Total** |
| --- | --- | --- | --- |
|  | **1** | **0** |  |
| **1** | 1 | 0 | 1 |
| **0** | 4 | 101 | 105 |
| **Total** | 5 | 101 | 106 |
|  | | | |
| Sensitivity % (95% CI) | Specificity % (95% CI) | PPV % (95% CI) | NPV % (95% CI) |
| 20 (1-72) | 100 (96-100) | 100 (3-100) | 96 (91-99) |

Malignancy

| **4 hour diagnosis** | **Final diagnosis** | | **Total** |
| --- | --- | --- | --- |
|  | **1** | **0** |  |
| **1** | 2 | 0 | 2 |
| **0** | 6 | 98 | 104 |
| **Total** | 8 | 98 | 106 |
|  | | | |
| Sensitivity % (95% CI) | Specificity % (95% CI) | PPV % (95% CI) | NPV % (95% CI) |
| 25 (3-65) | 100 (96-100) | 100 (16-100) | 94 (88-98) |

Others

| **4 hour diagnosis** | **Final diagnosis** | | **Total** |
| --- | --- | --- | --- |
|  | **1** | **0** |  |
| **1** | 19 | 10 | 29 |
| **0** | 14 | 63 | 77 |
| **Total** | 33 | 73 | 106 |
|  | | | |
| Sensitivity % (95% CI) | Specificity % (95% CI) | PPV % (95% CI) | NPV % (95% CI) |
| 58 (39-75) | 86 (76-93) | 66 (46-82) | 1. (71-90) |

*Too few ratings for the diagnostic accuracy calculations.
