# Supplementary material for: Point-of-care ultrasound of the heart and lungs in patients with respiratory failure: a pragmatic randomized controlled multicenter trial
Source: Scand J Trauma Resusc Emerg Med. 2021 Apr 26;29:60. doi: 10.1186/s13049-021-00872-8 (PMC8073910; doi:10.1186/s13049-021-00872-8)
Supplement: Supplementary file 5 — Additional file 5. [file 13049_2021_872_MOESM5_ESM.zip › Additional file 5b_ Diagnostic accuracy 4h_ctrl_cr1.docx]

**Additional file 5b**

**Diagnostic accuracy of 4 hour diagnoses. Control group.**

Correlation and diagnostic accuracy between investigators´ 4 hour presumptive diagnoses and final diagnoses (reference standards). Intention to treat population.

**Control group**

COPD in exacerbation

| **4 hour diagnosis** | **Final diagnosis** | | **Total** |
| --- | --- | --- | --- |
|  | **1** | **0** |  |
| **1** | 21 | 3 | 24 |
| **0** | 7 | 74 | 81 |
| **Total** | 28 | 77 | 105 |
|  | | | |
| Sensitivity % (95% CI) | Specificity % (95% CI) | PPV % (95% CI) | NPV % (95% CI) |
| 75 (55-89) | 96 (89-99) | 88 (68-97) | 91 (83-97) |

Asthma in exacerbation

| **4 hour diagnosis** | **Final diagnosis** | | **Total** |
| --- | --- | --- | --- |
|  | **1** | **0** |  |
| **1** | 2 | 1 | 3 |
| **0** | 1 | 101 | 102 |
| **Total** | 3 | 102 | 105 |
|  | | | |
| Sensitivity % (95% CI) | Specificity % (95% CI) | PPV % (95% CI) | NPV % (95% CI) |
| 67 (9-99) | 99 (94-100) | 67 (9-99) | 99 (94-100) |

Interstitial lung disease

| **4 hour diagnosis** | **Final diagnosis** | | **Total** |
| --- | --- | --- | --- |
|  | **1** | **0** |  |
| **1** | 0 | 2 | 2 |
| **0** | 5 | 98 | 103 |
| **Total** | 5 | 100 | 105 |
|  | | | |
| Sensitivity % (95% CI) | Specificity % (95% CI) | PPV % (95% CI) | NPV % (95% CI) |
| 0 (0-52) | 98 (93-100) | 0 (0-84) | 95 (89-98) |

Pneumonia

| **4 hour diagnosis** | **Final diagnosis** | | **Total** |
| --- | --- | --- | --- |
|  | **1** | **0** |  |
| **1** | 30 | 10 | 40 |
| **0** | 4 | 61 | 65 |
| **Total** | 34 | 71 | 105 |
|  | | | |
| Sensitivity % (95% CI) | Specificity % (95% CI) | PPV % (95% CI) | NPV % (95% CI) |
| 88 (73-97) | 86 (76-93) | 75 (59-87) | 94 (85-98) |

Pulmonary edema

| **4 hour diagnosis** | **Final diagnosis** | | **Total** |
| --- | --- | --- | --- |
|  | **1** | **0** |  |
| **1** | 2 | 2 | 4 |
| **0** | 4 | 97 | 101 |
| **Total** | 6 | 99 | 105 |
|  | | | |
| Sensitivity % (95% CI) | Specificity % (95% CI) | PPV % (95% CI) | NPV % (95% CI) |
| 33 (4-78) | 98 (93-100) | 50 (7-93) | 96 (90-99) |

Para-pneumonic effusion

| **4 hour diagnosis** | **Final diagnosis** | | **Total** |
| --- | --- | --- | --- |
|  | **1** | **0** |  |
| **1** | 5 | 2 | 7 |
| **0** | 9 | 89 | 98 |
| **Total** | 14 | 91 | 105 |
|  | | | |
| Sensitivity % (95% CI) | Specificity % (95% CI) | PPV % (95% CI) | NPV % (95% CI) |
| 36 (13-65) | 98 (92-100) | 71 (29-96) | 91 (83-96) |

Pulmonary empyema *

| **4 hour diagnosis** | **Final diagnosis** | | **Total** |
| --- | --- | --- | --- |
|  | **1** | **0** |  |
| **1** | 0 | 0 | 0 |
| **0** | 0 | 105 | 105 |
| **Total** | 0 | 105 | 105 |
|  | | | |
| Sensitivity % (95% CI) | Specificity % (95% CI) | PPV % (95% CI) | NPV % (95% CI) |
| **-** | - | - | - |

Pulmonary emboli

| **4 hour diagnosis** | **Final diagnosis** | | **Total** |
| --- | --- | --- | --- |
|  | **1** | **0** |  |
| **1** | 2 | 5 | 7 |
| **0** | 0 | 98 | 98 |
| **Total** | 2 | 103 | 105 |
|  | | | |
| Sensitivity % (95% CI) | Specificity % (95% CI) | PPV % (95% CI) | NPV % (95% CI) |
| 100 (16-100) | 96 (89-98) | 29 (4-71) | 100 (96-100) |

Pneumothorax

| **4 hour diagnosis** | **Final diagnosis** | | **Total** |
| --- | --- | --- | --- |
|  | **1** | **0** |  |
| **1** | 1 | 2 | 3 |
| **0** | 0 | 102 | 102 |
| **Total** | 1 | 104 | 105 |
|  | | | |
| Sensitivity % (95% CI) | Specificity % (95% CI) | PPV % (95% CI) | NPV % (95% CI) |
| 100 (3-100) | 98 (93-100) | 33 (1-91) | 100 (96-100) |

Systolic heart failure

| **4 hour diagnosis** | **Final diagnosis** | | **Total** |
| --- | --- | --- | --- |
|  | **1** | **0** |  |
| **1** | 13 | 5 | 18 |
| **0** | 9 | 78 | 87 |
| **Total** | 22 | 83 | 105 |
|  | | | |
| Sensitivity % (95% CI) | Specificity % (95% CI) | PPV % (95% CI) | NPV % (95% CI) |
| 59 (36-79) | 94 (87-98) | 72 (47-90) | 90 (81-95) |

Non-systolic heart failure

| **4 hour diagnosis** | **Final diagnosis** | | **Total** |
| --- | --- | --- | --- |
|  | **1** | **0** |  |
| **1** | 1 | 1 | 2 |
| **0** | 0 | 103 | 103 |
| **Total** | 1 | 104 | 105 |
|  | | | |
| Sensitivity % (95% CI) | Specificity % (95% CI) | PPV % (95% CI) | NPV % (95% CI) |
| 100 (3-100) | 99 (95-100) | 50 (1-99) | 100 (97-100) |

Acute myocardial infarction

| **4 hour diagnosis** | **Final diagnosis** | | **Total** |
| --- | --- | --- | --- |
|  | **1** | **0** |  |
| **1** | 1 | 8 | 9 |
| **0** | 1 | 95 | 96 |
| **Total** | 2 | 103 | 105 |
|  | | | |
| Sensitivity % (95% CI) | Specificity % (95% CI) | PPV % (95% CI) | NPV % (95% CI) |
| 50 (1-99) | 92 (85-97) | 11 (0-48) | 99 (94-100) |

Anemia

| **4 hour diagnosis** | **Final diagnosis** | | **Total** |
| --- | --- | --- | --- |
|  | **1** | **0** |  |
| **1** | 0 | 1 | 1 |
| **0** | 6 | 98 | 104 |
| **Total** | 6 | 99 | 105 |
|  | | | |
| Sensitivity % (95% CI) | Specificity % (95% CI) | PPV % (95% CI) | NPV % (95% CI) |
| 0 (0-46) | 99 (95-100) | 0 (0-98) | 94 (88-98) |

Malignancy

| **4 hour diagnosis** | **Final diagnosis** | | **Total** |
| --- | --- | --- | --- |
|  | **1** | **0** |  |
| **1** | 5 | 2 | 7 |
| **0** | 14 | 84 | 98 |
| **Total** | 19 | 86 | 105 |
|  | | | |
| Sensitivity % (95% CI) | Specificity % (95% CI) | PPV % (95% CI) | NPV % (95% CI) |
| 26 (9-51) | 98 (92-100) | 71 (29-96) | 86 (77-92) |

Others

| **4 hour diagnosis** | **Final diagnosis** | | **Total** |
| --- | --- | --- | --- |
|  | **1** | **0** |  |
| **1** | 21 | 10 | 31 |
| **0** | 14 | 60 | 74 |
| **Total** | 35 | 70 | 105 |
|  | | | |
| Sensitivity % (95% CI) | Specificity % (95% CI) | PPV % (95% CI) | NPV % (95% CI) |
| 60 (42-76) | 86 (75-93) | 1. (49-83) | 81 (70-89) |

*Too few ratings to perform diagnostic accuracy calculations.
