# Supplementary material for: Point-of-care ultrasound of the heart and lungs in patients with respiratory failure: a pragmatic randomized controlled multicenter trial
Source: Scand J Trauma Resusc Emerg Med. 2021 Apr 26;29:60. doi: 10.1186/s13049-021-00872-8 (PMC8073910; doi:10.1186/s13049-021-00872-8)
Supplement: Supplementary file 6 — Additional file 6. [file 13049_2021_872_MOESM6_ESM.zip › Additional file 6a Other diagnoses_int.docx]

**Additional file 6a : Other diagnoses or audit diagnoses that do not fulfill the diagnostic criteria.** Defined by medical record audit. Patients can also have other well defined final diagnoses along with these diagnoses. Per protocol population.

**Intervention group**

| **Patient ID** | **Most likely other diagnosis?** |  |
| --- | --- | --- |
| [122-1](https://open.rsyd.dk/redcap/redcap_v8.10.7/DataEntry/index.php?pid=183&id=122-1&page=randomisering) | Dyspepsia |  |
| [122-7](https://open.rsyd.dk/redcap/redcap_v8.10.7/DataEntry/index.php?pid=183&id=122-7&page=randomisering) | Influenza / upper respiratory tract infection. Psychogenic conditioned hyperventilation. |  |
| [122-10](https://open.rsyd.dk/redcap/redcap_v8.10.7/DataEntry/index.php?pid=183&id=122-10&page=randomisering) | Hyperventilation. Probably anxiety and depression. |  |
| [123-5](https://open.rsyd.dk/redcap/redcap_v8.10.7/DataEntry/index.php?pid=183&id=123-5&page=randomisering) | Post infectious coughing. |  |
| [123-10](https://open.rsyd.dk/redcap/redcap_v8.10.7/DataEntry/index.php?pid=183&id=123-10&page=randomisering) | Non-specific discomfort in the chest. |  |
| [123-20](https://open.rsyd.dk/redcap/redcap_v8.10.7/DataEntry/index.php?pid=183&id=123-20&page=randomisering) | Muscular conditional thoracic pain. |  |
| [123-30](https://open.rsyd.dk/redcap/redcap_v8.10.7/DataEntry/index.php?pid=183&id=123-30&page=randomisering) | Suspected heart failure, but does not meet the diagnostic criteria. |  |
| [123-35](https://open.rsyd.dk/redcap/redcap_v8.10.7/DataEntry/index.php?pid=183&id=123-35&page=randomisering) | * |  |
| [126-4](https://open.rsyd.dk/redcap/redcap_v8.10.7/DataEntry/index.php?pid=183&id=126-4&page=randomisering) | Upper airway tract infection |  |
| [126-12](https://open.rsyd.dk/redcap/redcap_v8.10.7/DataEntry/index.php?pid=183&id=126-12&page=randomisering) | Myosis |  |
| [126-13](https://open.rsyd.dk/redcap/redcap_v8.10.7/DataEntry/index.php?pid=183&id=126-13&page=randomisering) | Propable AHF, but does not meet the diagnostic criteria. |  |
| [126-28](https://open.rsyd.dk/redcap/redcap_v8.10.7/DataEntry/index.php?pid=183&id=126-28&page=randomisering) | Myoser |  |
| [126-39](https://open.rsyd.dk/redcap/redcap_v8.10.7/DataEntry/index.php?pid=183&id=126-39&page=randomisering) | Upper airway tract infection  Psycogenic hyperventilation |  |
| [128-5](https://open.rsyd.dk/redcap/redcap_v8.10.7/DataEntry/index.php?pid=183&id=128-5&page=randomisering) | (Chest pain and dyspnea: Chronic thromboembolic pulmonal hypertension is later diagnosed) |  |
| [128-8](https://open.rsyd.dk/redcap/redcap_v8.10.7/DataEntry/index.php?pid=183&id=128-8&page=randomisering) | Myoser |  |
| [128-16](https://open.rsyd.dk/redcap/redcap_v8.10.7/DataEntry/index.php?pid=183&id=128-16&page=randomisering) | Probably COLD in exacerbation, but does not meet the diagnostic critera. |  |
| [128-25](https://open.rsyd.dk/redcap/redcap_v8.10.7/DataEntry/index.php?pid=183&id=128-25&page=randomisering) | Psycogenic chest pain. |  |
| [128-31](https://open.rsyd.dk/redcap/redcap_v8.10.7/DataEntry/index.php?pid=183&id=128-31&page=randomisering) | Extrapleural abscess with fistula to pleura due to tuberculosis. |  |
| [128-38](https://open.rsyd.dk/redcap/redcap_v8.10.7/DataEntry/index.php?pid=183&id=128-38&page=randomisering) | Muscular conditional thoracic pain. |  |
| [128-39](https://open.rsyd.dk/redcap/redcap_v8.10.7/DataEntry/index.php?pid=183&id=128-39&page=randomisering) | Muscular conditional thoracic pain. |  |
| [129-4](https://open.rsyd.dk/redcap/redcap_v8.10.7/DataEntry/index.php?pid=183&id=129-4&page=randomisering) | Muscular conditional thoracic pain. |  |
| [129-13](https://open.rsyd.dk/redcap/redcap_v8.10.7/DataEntry/index.php?pid=183&id=129-13&page=randomisering) | * |  |
| [130-19](https://open.rsyd.dk/redcap/redcap_v8.10.7/DataEntry/index.php?pid=183&id=130-19&page=randomisering) | Muscular conditional thoracic pain |  |
| [130-32](https://open.rsyd.dk/redcap/redcap_v8.10.7/DataEntry/index.php?pid=183&id=130-32&page=randomisering) | * |  |
| [130-33](https://open.rsyd.dk/redcap/redcap_v8.10.7/DataEntry/index.php?pid=183&id=130-33&page=randomisering) | Anxiety attack |  |
| [130-40](https://open.rsyd.dk/redcap/redcap_v8.10.7/DataEntry/index.php?pid=183&id=130-40&page=randomisering) | Upper airway tract infection |  |
| [130-44](https://open.rsyd.dk/redcap/redcap_v8.10.7/DataEntry/index.php?pid=183&id=130-44&page=randomisering) | * |  |
| [130-49](https://open.rsyd.dk/redcap/redcap_v8.10.7/DataEntry/index.php?pid=183&id=130-49&page=randomisering) | Influenza |  |
| [130-58](https://open.rsyd.dk/redcap/redcap_v8.10.7/DataEntry/index.php?pid=183&id=130-58&page=randomisering) | * |  |
| [130-61](https://open.rsyd.dk/redcap/redcap_v8.10.7/DataEntry/index.php?pid=183&id=130-61&page=randomisering) | Atypical angina pectoris |  |
| [227-4](https://open.rsyd.dk/redcap/redcap_v8.10.7/DataEntry/index.php?pid=183&id=227-4&page=randomisering) | Hyperventilation |  |
|  |  |  |

* No description of other pathology or presumptive diagnoses
