# Supplementary material for: Point-of-care ultrasound of the heart and lungs in patients with respiratory failure: a pragmatic randomized controlled multicenter trial
Source: Scand J Trauma Resusc Emerg Med. 2021 Apr 26;29:60. doi: 10.1186/s13049-021-00872-8 (PMC8073910; doi:10.1186/s13049-021-00872-8)
Supplement: Supplementary file 6 — Additional file 6. [file 13049_2021_872_MOESM6_ESM.zip › Additional file 6b Other diagnoses_ctrl.docx]

**Additional file 6b. Other diagnoses or audit diagnoses that do not fulfill the diagnostic criteria.** Defined by medical record audit. Patients can also have other well defined final diagnoses along with these diagnoses. Per protocol population.

**Control**

| **Patient ID** | **Most likely other diagnosis?** |  |
| --- | --- | --- |
| [123-1](https://open.rsyd.dk/redcap/redcap_v8.10.7/DataEntry/index.php?pid=183&id=123-1&page=randomisering) | Muscular conditional thoracic pain |  |
| [123-3](https://open.rsyd.dk/redcap/redcap_v8.10.7/DataEntry/index.php?pid=183&id=123-3&page=randomisering) | Pickwick syndrome |  |
| [123-9](https://open.rsyd.dk/redcap/redcap_v8.10.7/DataEntry/index.php?pid=183&id=123-9&page=randomisering) | Muscular pain |  |
| [123-22](https://open.rsyd.dk/redcap/redcap_v8.10.7/DataEntry/index.php?pid=183&id=123-22&page=randomisering) | Muscular conditional thoracic pain |  |
| [123-28](https://open.rsyd.dk/redcap/redcap_v8.10.7/DataEntry/index.php?pid=183&id=123-28&page=randomisering) | Drug induced adverse events (Capecitabine) |  |
| [123-32](https://open.rsyd.dk/redcap/redcap_v8.10.7/DataEntry/index.php?pid=183&id=123-32&page=randomisering) | Interstitial lung disease |  |
| [123-33](https://open.rsyd.dk/redcap/redcap_v8.10.7/DataEntry/index.php?pid=183&id=123-33&page=randomisering) | Muscular conditional thoracic pain |  |
| [126-3](https://open.rsyd.dk/redcap/redcap_v8.10.7/DataEntry/index.php?pid=183&id=126-3&page=randomisering) | Malignant pleural effusion |  |
| [126-9](https://open.rsyd.dk/redcap/redcap_v8.10.7/DataEntry/index.php?pid=183&id=126-9&page=randomisering) | Muscular conditional thoracic pain |  |
| [126-10](https://open.rsyd.dk/redcap/redcap_v8.10.7/DataEntry/index.php?pid=183&id=126-10&page=randomisering) | Cholangitis |  |
| [126-11](https://open.rsyd.dk/redcap/redcap_v8.10.7/DataEntry/index.php?pid=183&id=126-11&page=randomisering) | Gastroenteritis |  |
| [126-25](https://open.rsyd.dk/redcap/redcap_v8.10.7/DataEntry/index.php?pid=183&id=126-25&page=randomisering) | Hypoglycemia |  |
| [126-29](https://open.rsyd.dk/redcap/redcap_v8.10.7/DataEntry/index.php?pid=183&id=126-29&page=randomisering) | Pleuritis |  |
| [128-2](https://open.rsyd.dk/redcap/redcap_v8.10.7/DataEntry/index.php?pid=183&id=128-2&page=randomisering) | Pulmonal hypertension (+ bilateral pleural effusion (er registreret)) |  |
| [128-4](https://open.rsyd.dk/redcap/redcap_v8.10.7/DataEntry/index.php?pid=183&id=128-4&page=randomisering) | Pleuritis |  |
| [128-7](https://open.rsyd.dk/redcap/redcap_v8.10.7/DataEntry/index.php?pid=183&id=128-7&page=randomisering) | Cirrosis |  |
| [128-10](https://open.rsyd.dk/redcap/redcap_v8.10.7/DataEntry/index.php?pid=183&id=128-10&page=randomisering) | Pancreatitis |  |
| [128-33](https://open.rsyd.dk/redcap/redcap_v8.10.7/DataEntry/index.php?pid=183&id=128-33&page=randomisering) | Upper airway tract infection |  |
| [128-36](https://open.rsyd.dk/redcap/redcap_v8.10.7/DataEntry/index.php?pid=183&id=128-36&page=randomisering) | Hypersensitivity pneumonitis (probable exacerbation in interstitial lung disease) |  |
| [129-5](https://open.rsyd.dk/redcap/redcap_v8.10.7/DataEntry/index.php?pid=183&id=129-5&page=randomisering) | * |  |
| [129-6](https://open.rsyd.dk/redcap/redcap_v8.10.7/DataEntry/index.php?pid=183&id=129-6&page=randomisering) | Muscular conditional thoracic pain |  |
| [129-9](https://open.rsyd.dk/redcap/redcap_v8.10.7/DataEntry/index.php?pid=183&id=129-9&page=randomisering) | Urinary tract infection |  |
| [129-10](https://open.rsyd.dk/redcap/redcap_v8.10.7/DataEntry/index.php?pid=183&id=129-10&page=randomisering) | Pneumonia, but does not meet the diagnostic criteria |  |
| [130-2](https://open.rsyd.dk/redcap/redcap_v8.10.7/DataEntry/index.php?pid=183&id=130-2&page=randomisering) | Hyponatremia |  |
| [130-4](https://open.rsyd.dk/redcap/redcap_v8.10.7/DataEntry/index.php?pid=183&id=130-4&page=randomisering) | Arthritis urica |  |
| [130-9](https://open.rsyd.dk/redcap/redcap_v8.10.7/DataEntry/index.php?pid=183&id=130-9&page=randomisering) | Emfysema |  |
| [130-10](https://open.rsyd.dk/redcap/redcap_v8.10.7/DataEntry/index.php?pid=183&id=130-10&page=randomisering) | Urinary tract infection |  |
| [130-31](https://open.rsyd.dk/redcap/redcap_v8.10.7/DataEntry/index.php?pid=183&id=130-31&page=randomisering) | Cholecystolithiasis |  |
| [130-43](https://open.rsyd.dk/redcap/redcap_v8.10.7/DataEntry/index.php?pid=183&id=130-43&page=randomisering) | Non-systolic HF – does not meet the diagnostic |  |
| [130-47](https://open.rsyd.dk/redcap/redcap_v8.10.7/DataEntry/index.php?pid=183&id=130-47&page=randomisering) | Upper airway tract infection |  |
| [130-48](https://open.rsyd.dk/redcap/redcap_v8.10.7/DataEntry/index.php?pid=183&id=130-48&page=randomisering) | Osteomyelitis |  |
| [227-3](https://open.rsyd.dk/redcap/redcap_v8.10.7/DataEntry/index.php?pid=183&id=227-3&page=randomisering) | Uremia and metabolic acidosis |  |
| [227-6](https://open.rsyd.dk/redcap/redcap_v8.10.7/DataEntry/index.php?pid=183&id=227-6&page=randomisering) | Systolic HF – does not meet the diagnostic criteria |  |
| [227-9](https://open.rsyd.dk/redcap/redcap_v8.10.7/DataEntry/index.php?pid=183&id=227-9&page=randomisering) | Muscular pain |  |
| [227-12](https://open.rsyd.dk/redcap/redcap_v8.10.7/DataEntry/index.php?pid=183&id=227-12&page=randomisering) | Malignancy, Systolic HF, pulmonary edema, urosepsis, bacteraemia – but does not meet the diagnostic criteria of these. |  |

* No description of other pathology or presumptive diagnoses
