# Supplementary material for: Point-of-care ultrasound of the heart and lungs in patients with respiratory failure: a pragmatic randomized controlled multicenter trial
Source: Scand J Trauma Resusc Emerg Med. 2021 Apr 26;29:60. doi: 10.1186/s13049-021-00872-8 (PMC8073910; doi:10.1186/s13049-021-00872-8)
Supplement: Supplementary file 7 — Additional file 7. [file 13049_2021_872_MOESM7_ESM.docx]

**Additional file 7**

**Cause of death in patients, who died within 30 days from admission. Per protocol population.**

| **Cause of death** | **n** |
| --- | --- |
| Respiratory insufficiency | 6 |
| Others | 1 |

Due to general data protection regulation numbers n <5 will not be classified.
