# Supplementary material for: Point-of-care ultrasound of the heart and lungs in patients with respiratory failure: a pragmatic randomized controlled multicenter trial
Source: Scand J Trauma Resusc Emerg Med. 2021 Apr 26;29:60. doi: 10.1186/s13049-021-00872-8 (PMC8073910; doi:10.1186/s13049-021-00872-8)
Supplement: Supplementary file 8 — Additional file 8. [file 13049_2021_872_MOESM8_ESM.docx]

**Table 1:** Baseline characteristics (per protocol population).

|  | Intervention group (n= 102 ) | Control group (n=99 ) |
| --- | --- | --- |
| Age (years; median (IQR) | 68 (52-79) | 72 (60-80) |
| Sex  Male  Female | 60 (58.8%)  42 (41%) | 42 (42%)  57 (58%) |
| Smoking status  Never smoked  Current smoker  Previous smoker  Unknown status | 28 (27%)  26 (25%)  24 (24%)  24 (24%) | 21 (21%)  10 (10%)  37 (37%)  31 (31%) |
| Medical history  Apoplexy  Coronary artery disease  Heart failure  Arterial hypertension  Thromboembolic disease  Chronic obstructive pulmonary disease  Asthma  Other pulmonary or pleural lung disease  Diabetes mellitus  Chronic kidney disease  Other medical disease  Psychiatric disorder | 9 (9%)  16 (16%)  13 (13%)  22 (22%)  11 (11%)  32 (31%)  5 (5%)  11 (11%)  7 (7%)  8 (8%)  28 (27%)  7 (7%) | 7 (7%)  16 (16%)  14 (14%)  20 (20%)  5 (5%)  30 (30%)  6 (6%)  9 (9%)  17 (17%)  8 (8%)  36 (36%)  3 (3%) |
| Medication at admission  Β-blockers  Diuretics  Nitrates  Angiotensin-converting-enzyme inhibitor  or angiotensin-receptor blocker  Digoxin  Calcium-channel blockers  Aspirin  Inhaled bronchodilators  Inhaled corticosteroids  Oral corticosteroids  Antibiotics  Anticoagulants  Immunosuppressive medication  Other medication | 28 (27%)  37 (36%)  9 (9%)  24 (24%)  4 (4%)  16 (16%)  16 (16%)  38 (37%)  21 (21%)  9 (9%)  17 (17%)  27 (26%)  5 (5%)  18 (18%) | 21 (21%)  47 (47%)  14 (14%)  37 (37%)  5 (5%)  20 (20%)  18 (18%)  42 (42%)  28 (28%)  17 (17%)  15 (15%)  34 (34%)  2 (2%)  22 (22%) |
| Vital signs at admission mean. (min-max)  Respiratory rate (breaths per min)  Saturation (%)   - of these patients received oxygen supply n (l/min)   - 1 – 3 l/min n (%)   - 4-6 l/min n (%)   - > 6 l/min n (%)   Systolic blood pressure (mmHg)  Diastolic blood pressure (mmHg)  Heart rate (beats per minute)  Temperature (°C)  Blood glucose  Glasgow coma scale score^a^ | 22 (12-44)  94 (55-100)  23 (1-6 l/min)  12 (52%)  8 (35%)  0  137 (80-224)  79 (68-86)  90 (52-150)  37.1 (35.2-39.5)  6.6 (4.1-12)  15 (9-15) | 22 (12-35)  95 (74-100)  27 (1-12 l/min)  22 (81)  4 15)  1 (4)  136 (95-212)  78 (47-111)  86 (40-144)  37.1 (35.0-39.8)  7.0 (3.7-22.4)  15 (14-15) |
| Patients´ signs and symptoms upon admission  Cough  Dyspnoea  Chest pain  Respiration rate > 20 breaths per min  Peripheral saturation < 95%  None of the above  Ultrasound examination already performed in ambulance | 30 (29%)  87 (85%)  26 (25%)  32 (31%)  29 (28%)  0  0 | 40 (40%)  81 (82%)  27 (27%)  29 (29%)  23 (23%)  0  0 |
| **Patients severity score upon admission**^b^   - I (Red) - II (Orange) - III (Yellow) - IV (Green) | 5 (5%)  29 (28%)  47 (46%)  21 (21%) | 0  33 (33%)  40 (40%)  26 (26%) |

Data are number(%).mean (SD) or a median (IQR). unless otherwise indicated.

Data are not available for all randomized patients. Missing data are handled by multiple imputation for continuous data and simple imputation when binominary.

^a^We found GCS 9 in one patient admitted with exacerbation in terminal COPD. Pt was immediately treated with NIV with effect, replied relevant on questions and signed informed consent. The remaining of the included patients had GCS from 14 - 15.

^b^  The severity score is made according to the Danish Emergency Process Triage (DEPT) criteria used for patients with acute illness. The severity score is assessed by measuring the patients´ vital parameters (e.g. BP. HR. GCS). `I (red)´ is the most severe condition.

**Table 2.** Final diagnoses and the proportion of presumptive diagnoses in agreement with final diagnoses.

|  | **Diagnoses in the intervention group (n= 102)** | | **Diagnoses in the control group (n=99)** | |
| --- | --- | --- | --- | --- |
| **Lungs** | Final diagnoses n (%) | Number of 4h presumptive diagnoses in agreement with final diagnoses, n (%). | Final diagnoses n (%) | Number of 4h presumptive diagnoses in agreement with final diagnoses, n (%). |
| Exacerbation of chronic obstructive lung disease | 26 (25%) | 24 (92%) | 27 (27%) | 20 (74%) |
| Asthma with exacerbation | 2 (2%) | 0 | 3 (3%) | 2 (67%) |
| Exacerbation in interstitial lung disease | 3 (3%) | 1 (33%) | 5 (5%) | 0 |
| Pneumonia | 25 (25%) | 22 (88%) | 30 (30%) | 28 (93%) |
| Pulmonary edema | 17 (17%) | 9 (53%) | 6 (6%) | 2 (33%) |
| Parapneumonic effusion | 13 (13%) | 10 (77%) | 13 (13%) | 5 (38%) |
| Empyema | 1 (1%) | 0 | 0 | 0 |
| Pulmonary embolism | 3 (3%) | 3 (100%) | 2 (2%) | 2 (100%) |
| Pneumothorax | 0 | 0 | 1 (1%) | 1 (100%) |
| **Heart** |  |  |  |  |
| Systolic heart failure | 17 (17%) | 13 (76%) | 21 (21%) | 13 (62%) |
| Non-systolic heart failure | 5 (5%) | 3 (60%) | 1 (1%) | 1 (100%) |
| Acute myocardial infarction | 1 (1%) | 0 | 1 (1%) | 1 (100%) |
| **Miscellaneous** |  |  |  |  |
| Anemia | 5 (5%) | 1 (20%) | 5 (5%) | 0 |
| Malignancy | 8 (8%) | 2 (25%) | 19 (19%) | 5 (26%) |
| No diagnostic criteria met  Of these x had  -chest pain for various reasons n (%)  -suspected of AHF *.** | 31 (30%)  9 (29%)  2 (6%) | 18 (58%) | 35 (35%)  5 (19%)  0 | 21 (60%) |

Per protocol population

Data are number (%) unless otherwise indicated.

*AHF: Acute heart failure. **Did not meet the diagnostic criteria

Legend: Patients in whom the defined audit diagnoses were established at the 4 hour assessment.

**Table 3.** Diagnostic accuracy of emergency physicians´ 4 hour presumptive diagnoses compared to final diagnoses. x

|  | **Final diagnosis positive / 4 hour positive** | | **Sensitivity % (95% CI)** | | **Specificity % (95% CI)** | | **PPV**  **% (95% CI)** | | **NPV**  **% (95% CI)** | |
| --- | --- | --- | --- | --- | --- | --- | --- | --- | --- | --- |
| **Diagnoses** | Interv. | Control | Interv. | Control | Interv. | Control | Interv. | Control | Interv. | Control |
| **COPD with exacerbation** | 26/29 | 27/23 | 92 (75-99) | 74 (54-89) | 93 (85-98) | 96 (88-99) | 83 (64-94) | 87 (66-97) | 97 (91-100) | 91 (82-96) |
| **Asthma with exacerbation** | 2/1 | 3/3 | 0 (0-84) | 67 (9-99) | 99 (95-100) | 99 (94-100) | 0 (0-98) | 67 (9-99) | 98 (93-100) | 99 (94-100) |
| **Interstitial lung disease** | 3/1 | 5/2 | 33 (0.8-91) | 0 (0-52) | 98 (93-100) | 98 (93-100) | 33 (1-91) | 0 (0-84) | 100 (97-100) | 95 (88-98) |
| **Pneumonia** | 25/32 | 30/38 | 88 (69-98) | 93 (78-99) | 87 (77-94) | 86 (75-93) | 69 (50-84) | 74 (57-87) | 96 (88-99) | 97 (89-100) |
| **Pulmonary edema** | 17/10 | 6/4 | 53 (28-77) | 33 (4-78) | 99 (94-100) | 98 (92-100) | 90 (56-100) | 50 (7-93) | 91 (84-96) | 96 (90-99) |
| **Para-pneumonic effusion** | 13/14 | 13/6 | 77 (46-95) | 39 (14-68) | 96 (89-99) | 99 (94-100) | 71 (42-92) | 83 (36-100) | 97 (90-99) | 91 (84-96) |
| **Pleural empyema*** | 1/0 | 0/0 |  |  |  |  |  |  |  |  |
| **Pulmonary embolism** | 3/11 | 2/6 | 100 (29-100) | 100 (16-100) | 92 (85-96) | 96 (90-99) | 27 (6-61) | 33 (4-78) | 100 (96-100) | 100 (96-100) |
| **Pneumothorax*** | 0/0 | 1/3 |  | 100 (2.5-100) |  | 98 (93-100) |  | 33 (0.8-91) |  | 100 (96-100) |
| **Systolic heart failure** | 17/16 | 21/18 | 77 (50-93) | 62 (38-82) | 97 (90-99) | 94 (86-98) | 81 (54-96) | 72 (47-90) | 95 (89-99) | 90 (82-96) |
| **Non-systolic heart failure** | 5/6 | 1/2 | 60 (15-95) | 100 (2.5-100) | 97 (91-99) | 99 (94-100) | 50 (12-88) | 50 (1.3-99) | 98 (93-100) | 100 (96-100) |
| **Acute myocardial infarction** | 1/2 | 1/9 | 0 (0-98) | 100 (2.5-100) | 98 (93-100) | 92 (85-96) | 0 (0-84) | 11 (0.3-48) | 99 (95-100) | 100 (96-100) |
| **Anemia** | 5/1 | 5/1 | 20 (0.5-72) | 0 (0-52) | 100 (96-100) | 99 (94-100) | 100 (2.5-100) | 0 (0-98) | 96 (90-99) | 95 (86-98) |
| **Malignancy** | 8/2 | 19/7 | 25 (3-65) | 26 (9-51) | 94 (96-100) | 98 (91-100) | 100 (16-100) | 71 (29-96) | 94 (87-98) | 85 (76-91) |
| **Other diagnoses** | 31/27 | 35/28 | 58 (39-76) | 60 (42-76) | 87 (77-94) | 89 (79-96) | 67 (46-84) | 75 (55-89) | 83 (72-90) | 80 (69-89) |

Per protocol population.

Intervention (n=102), control (n=99).

Abbreviation: Interv. (Intervention)

*Too few ratings to perform diagnostic accuracy calculations.

**Table 4.** Primary and secondary endpoints for the per protocol population.

|  | **Intervention group (n=102)** | **Control group (n=99)** | **P value** | **Absolute effect %**  **(95% CI)** | **Relative effect**  **(95% CI)** |
| --- | --- | --- | --- | --- | --- |
| **PRIMARY ENDPOINT** |  |  |  |  |  |
| **4h after admission to the emergency department**  Patients with presumptive diagnoses in agreement with final diagnoses | 81 (79.4%; 70.3-86.3) | 78 (78.8%; 69.5-85.8) | 0.91 | 0.6% (-0.1-0.1) | - 1. (0.87-1.16) |
| **SECONDARY ENDPOINTS** |  |  |  |  |  |
| **4h after admission to the emergency department**  Appropriate treatment ordered* | 81 (79.4%; 70.3-86.3) | 65 (65.7%; 55.6-74.5) | 0.03 | 13.8% (0.0-0.3) | 1.21 (1.02-1.44) |
| **After primary assessment in the emergency department**  Patients with presumptive diagnoses in agreement with final diagnoses | 63 (61.8%; 51.8-70.8) | 62 (62.6%; 52.5-71.7) | 0.90 | -0.9% (-0.1-0.1) | 0.98(0.79-1.22) |
| **Specific treatment ordered within 4h from admission to the emergency department**  Oxygen  NIV/CPAP  Respirator  Bronchodilators  Systemic steroids  Antibiotics  Fluids i.v.  Diuretics  Antiarrythmics  Vasoconstrictors  Anticoagulants  Therapeutic centesis ^b^  Others | 32 (31.4%; 22.5-41.3)  3 (2.94%; 0.6-8.4)  0 (0%; 0-3.6¥)  25 (24.5%; 16.5-34.0)  23 (22.5%; 14.9-31.9)  29 (28.4%; 19.9-38.2)  14 (13.7%; 7.7-22.0)  13 (12.7%; 7.0-20.8)  4 (3.9%; 1.1-9.7)  0 (0%; 0-3.5¥)  10 (9.8%; 4.8-17-3)  3 (2.9%; 0.6-8.4)  8 (7.8%; 3.4-14.9) | 35 (35.4%; 26.0-45.6)  0 (0%; 0-3.7¥)  0 (0%; 0-3.7¥)  19 (19.2%; 12.0-28.3)  20 (20.2%; 12.8-29-5)  35 (35.4%; 26.0-45.6)  23 (23.3%; 15.3-32.8)  11 (11.1%; 5.7-19.0)  3 (3.0%; 0.6-8.6)  0 (0%; 0-3.7¥)  10 (10.1%; 5.0-17.8)  3 (3.0%; 0.6-8.6)  6 (6.1%; 2.3-12.7) | 0.55  0.09  -  0.36  0.69  0.29  0.08  0.72  0.73  -  0.91  0.97  0.61 | -4.0% (-16.4-8.4)  2.3% (0.0-1.4)  0  5.0% (-5.9-15.9)  2.2% (-8.6-13.1)  -7.7% (-20.0-4.6)  -8.8% (-19.0-1.4)  1.1% (-7.3-9.6)  0.1% (3.7-5.7)  0  -3.5% (-11.8-4.8)  -0.1% (-4.3-4.4)  2.0 (-4.6-8.5) | 0.89 (0.60.0-1.31)  -  -  1.28(0.75-2.17)  1.11 (0.66-1.90)  0.80 (0.54-1.20)  0.59 (0.32-1.08)  1.14 (0.53-2.43)  1.29 (0.28-5.63)  -  0.97 (0.42-2.23)  0.97 (0.20-4.69)  1.29 (0.47-3.60) |
| **Supplementary diagnostic tests ordered within 4 h after admission to the ED**  Ultrasound of the lungs ^c^  X-ray of the thorax  CT of the thorax  MR of the thorax  Diagnostic centesis^b^  Echocardiography by cardiologist  Ultrasound of the deep veins ^d^  Other diagnostic tests | 33 (32.4%; 0.2-0.4)  82 (80.4%; 0.71-0.9)  10 (9.8%; 0.0-0.2)  0 (0%; 0-3.6¥)  1 (1.0%; 0.0-0.1)  15 (14.7%; 0.1-0.2)  2 (2.0%; 0.0-0.1)  23 (22.5%; 15.9-31.9) | 7 (7.1%; 2.9-14.0 )  84 (84.8%; 76.2-91.3)  9 (9.1%; 4.2-16.6)  0 (0%; 0-3.7¥)  2 (2.0%; 0.2-7.1 )  15 (15.2%; 8.7-23.8)  0 (0%; 0-3.7¥)  10 (10.1%; 5.0-17.8) | 0.00  0.40  0.85  -  0.54  0.92  0.16  0.02 | 22.4% (12.1-32.7)  -4.0% (-14.5-6.4)  1.1% (-6.-8.5)  0  -0.9% (-4.0-2.2)  -3.4% (-13.0-6.2)  1.9% (-0.7-4.4)  11.3% (1.5-21.1) | 4.58 (2.12-9.86)  0.95 (0.83-1.08)  1.08 (0.46-2.54)  -  0.49 (0.04-5.27)  0.97 (0.50-1.88)  -  2.23 (1.12-4.45) |
| **Time spent in the ED (hours)**  <1  1-2  3-3  5-8  9-24  25-48  >48 | 23 (22.6%; 15.4-31.8)  19 (18.6%; 12.1-27.5)  23 (22.6%; 15.4-31.8)  14 (13.7%; 8.2-22.0)  9 (8.8%; 4.6-16.3)  8 (7.8%; 3.9-15.1)  6 (5.9%; 2.6-12.6) | 25 (25.3%; 17.6-35.0)  24 (24.2%; 16.7-33.8)  18 (18.2%; 11.7-27.2)  16 (16.2%; 10.1-24.9)  5 (5.1%; 2.1-11.7)  5 (5.1%; 2.1-11.7)  7 (7.1%; 3.4-14.3) | 0.65  0.33  0.44  0.68  0.29  0.42  0.96 | -2.7% (-14.5-9.09)  -5.6% (-16.9-5.7)  4.4% (-6.7-15.5)  -2.4% (-12.3-7.4)  3.8% (-3.2- 10.8)  2.7% (-4.0-9.6)  -0.2% (-6.7-6.4) | 0.89 (0.54-1.46)  0.77 (0.45-1.31)  1.24 (0.71-2.15)  0.85 (0.44-1.65)  1.75 (0.61-5.03)  1.55 (0.53-4.58)  0.97 (0.32-2.91) |
| **Time spent in hospital (days)**  <1  1  2  3  4-7  >7 | 38 (37.3%; 28.3-47.2)  14 (13.7%;8.2-22.0)  8 (7.8%;3.9-15.1)  11 (10.8%;6.0-18.6)  16 (15.7%;9.8-24.3)  15 (14.7%;9.0-23.1) | 25 (25.3%; 17.6-34.9)  21 (21.2%;14.2-30.5)  9 (9.1%; 4.7-16.7)  6 (6.1%; 2.7-13.0)  22 (22.2%;15.0-31.6)  16 (16.2%;10.1-24.9) | 0.07  0.16  0.75  0.23  0.24  0.78 | 12.0% (-0.7-24.7)  -7.4% (-17.9-3.0)  -1.2% (-8.9-6.4)  4.7% (-2.9-12.4)  -6.5 (-17.3-4.3)  -1.4 (-11.4- 8.5) | 1.48 (0.97-2.25)  0.65 (0.35-1.20)  0.86 (0.35-2.15)  1.78 (0.68-4.63)  0.71 (0.39-1.26)  0.91 (0.48-1.74) |
| **The patients´ itinerary: After discharge from the emergency department patients were**  Transferred to the ICU  Transferred to a hospital ward  Sent home | 0 (0%; 0-3.6¥)  51 (50.0%; 40.3-59.7)  51 (50.0%; 40.3-59.7) | 1 (1%; 0.1-7.0)  47 (47%; 37.7-57.5)  51 (52%; 41.6-61.3) | 0.31  0.72  0.83 | -1.0% (-0.30-0.01)  2.5% (-0.11-0.16)  -1.5% (-0.15-0.12) | 0  1.05 (0.79-1.40)  0.97 (0.74-1.27) |
| **Readmission**  Patients readmitted ≤30 days from discharge | 23 (22.5%; 15.4-31.8) | 22 (22.2%; 15.0-31.6) | 0.96 | 0.3% (-11.2-11.9) | 1.01 (0.61-1.70) |
| **Mortality**  In hospital  30 day mortality | 2 (2.0%; 0.2-6.9)  2 (2.0%; 0.2-6.9) | 3 (3.0%; 0.6-8.6)  6 (6.1%; 2.3-12.7) | 0.63  0.14 | -1-1% (-5.4-3.2)  -4.1% (-9.5-1.3) | 0.64 (0.11-3.79)  0.32 (0.07-1.56) |

**Abbreviations:** n (number of patients). NIV (Non-invasive ventilation). CPAP (Continuous positive airway pressure). ED (emergency department). ICU (intensive care unit). ^¥^ One-side. 97.5% confidence interval. ^a^According to local treatment guidelines for the diseases in question. ^b^ Pleura-. thoraco-. cardio-centesis. ^c^ Performed by a radiologist or ultrasonographer certified in lung ultrasound. ^d^ Performed by radiologist or certified ultrasonographer.
